# From coral reefs into the abyss: the evolution of corallivory in the Coralliophilinae (Neogastropoda, Muricidae)

**DOI:** 10.1007/s00338-024-02537-1

**Published:** 2024-08-12

**Authors:** Elisa Nocella, Giulia Fassio, Dario Zuccon, Nicolas Puillandre, Maria Vittoria Modica, Marco Oliverio

**Affiliations:** 1 Department of Biology and Biotechnologies “Charles Darwin”, Sapienza University of Rome, Rome, Italy; 2 Department of Biology and Evolution of Marine Organisms, Stazione Zoologica Anton Dohrn, Naples, Italy; 3 Institut Systématique Evolution Biodiversité (ISYEB), Muséum National d’Histoire Naturelle, CNRS, Sorbonne Université, EPHE, Université des Antillles, 57 Rue Cuvier, CP 51, 75005 Paris, France

**Keywords:** Host–parasite interactions, Corallivory, Cnidaria, Gastropods, Coevolution, Molecular phylogeny

## Abstract

In this study, we delved into the interaction between corallivorous marine gastropods, the muricid Coralliophilinae Chenu, 1859, and their cnidarian food targets. Coralliophilinae is a subfamily of specialised corallivorous caenogastropods that feed by browsing on octocorals or hexacorals. Only sparse information is available on the phylogenetic relationships and the degree of specificity of the trophic relationships within this corallivorous lineage. To address these gaps, we generated the largest molecular dataset to date, comprising two mitochondrial (*cox1* and *16S* rDNA) and one nuclear gene (*ITS2* rDNA) from 586 specimens collected worldwide. The coral hosts of coralliophilines were identified through an integrative approach, combining literature data with new records, employing morphological and/or molecular markers, and incorporating data from DNA barcoding of the snail stomach content. Our comprehensive approach unveiled the existence of numerous cryptic species in Coralliophilinae, while the phylogeny showed that most of the currently accepted genera are not monophyletic. The molecular dating confirmed the origin of the Coralliophilinae in Middle Eocene, with diversification of most lineages during the Miocene. Our results indicate that the subfamily’s ancestor evolved in shallow waters in association with Scleractinia. Through the evolutionary history of Coralliophilinae, multiple host shifts to other cnidarian orders were observed, not correlated with changes in the depth range. The results of diversification analyses within the subfamily further suggest that the association with the host has influenced the evolutionary patterns of Coralliophilinae, but not vice versa.

**Supplementary Information:**

The online version contains supplementary material available at 10.1007/s00338-024-02537-1.

## Introduction

Symbiotic interactions play crucial roles in marine ecosystems and are fundamental in shaping community dynamics (Pita et al. 2018). Asymmetric symbiotic interactions (such as host–parasite) may involve co-evolutionary patterns characterised by a delicate balance of adaptations and counter-adaptations between host and parasite (Van Der Laan and Hogeweg 1997) or reflect a sequential evolution mechanism (Jermy 1976), where the evolution of the host influences the evolution of the parasite, but not vice versa. Exploring the evolution of symbiosis necessitates reliable data on the associations as well as a robust systematic and phylogenetic framework of the groups involved in such associations.

**Fig. 1:**
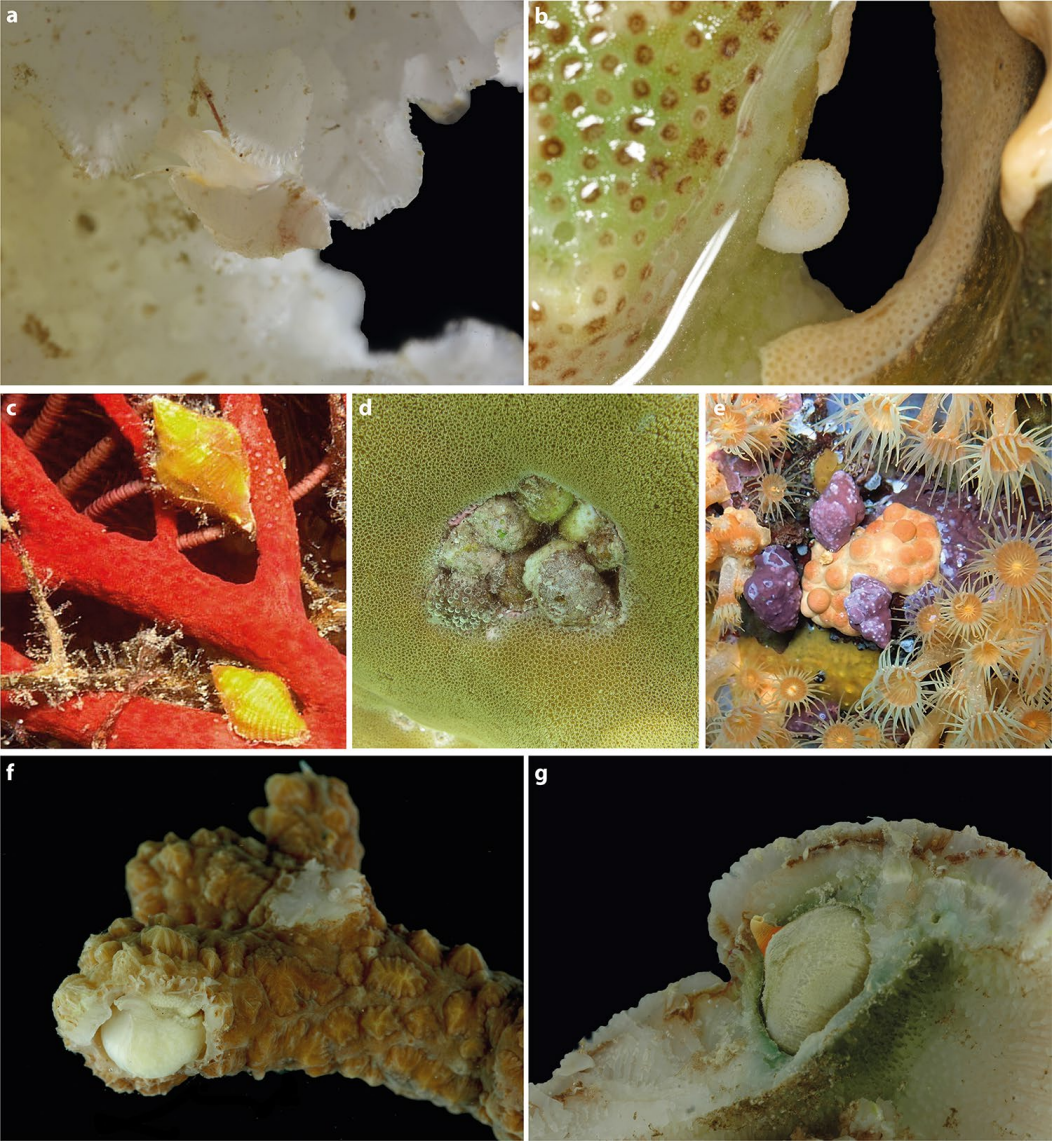
Living specimens of coralliophiline species on their hosts. **a** *Galeropsis monodontus* on Pocilloporidae, Papua New Guinea. **b** *Coralliophila radula* on Poritidae, New Caledonia. **c** *Coralliophila rubrococcinea* on Gorgoniidae, Philippines. **d** *Coralliophila violacea* on Poritidae, Kenya. **e** *Coralliophila meyendorffii* on Parazoanthidae, Italy. **f** *Leptoconchus* sp. in Merulinidae, Vanuatu. **g** *Leptoconchus* sp. in Fungiidae, Vanuatu. Photograph credits: **a** Laurent Charles (MNHN); **b** Philippe Maestrati (MNHN); **c** Guido Poppe (http://www.www.poppe-images.com); **d**–**e** Paolo Mariottini (University of Roma Tre); **f**–**g** Anne-Lise Fleddum (MNHN)

Coral reefs have been recognised as exceptionally diverse ecosystems, sustaining multiple symbiotic interactions (Stella et al. 2011). The vulnerability of cnidarians to climate change implies that the threats currently affecting oceans could not only affect their global decline but also have profound repercussions on the associated fauna (Pandolfi et al. 2003). Various gastropod groups have evolved the ability to feed on corals, despite their inherent toxicity, providing intriguing models for studying the evolutionary ecology of the association with corals. In particular, the muricid subfamily Coralliophilinae Chenu, 1859, is a highly diverse lineage of Neogastropods, currently comprising 268 extant species (WoRMS 2024). Coralliophilinae are distributed worldwide, mostly in warm temperate and tropical oceans (Oliverio et al. 2009b). All species for which the ecology is known exhibit symbiotic relationships (ecto or endobiotic) with anthozoans, including sea-anemones, soft corals, and scleractinians, on which they feed (Fig. 1). While some snail species feed on solitary polyps, others are associated with colonial anthozoans, with exceptional cases of species able to swap between solitary and colonial hexacorals (Oliverio 2008; Oliverio and Mariottini 2001).

For some coralliophiline species, significant impact of their trophic habits on coral reef communities has been reported (Hayes and Bush 1990). However, limited information is so far available regarding the level of specificity and the adaptive mechanisms underlying coralliophiline-cnidarian interactions (e.g. Oliverio et al. 2009a, b). While common in shallow waters, Coralliophilinae are better represented in the deep-water ecosystems, particularly in the mesophotic zone, but also in bathyal and abyssal habitats. Although Oliverio et al. (2009b) suggested that coralliophilines might have repeatedly invaded deep habitats, a formal analysis of the ancestral habitat for the subfamily has not been conducted. The monophyly of Coralliophilinae is strongly supported and their most probable sister groups has been identified in the Rapaninae and Ergalataxinae (Russini et al. 2023). However, the clarification of the evolutionary relationships within the subfamily has been complicated by the remarkable interspecific variation of Coralliophilinae shell features (Richter and Luque 2002). Preliminary molecular studies of Coralliophilinae phylogenetic relationships (Oliverio and Mariottini 2001; Oliverio et al. 2002a, b, 2009a) were penalised by a limited taxon sampling.

To establish a robust phylogenetic framework for taxonomic and macroevolutionary investigations of the subfamily Coralliophilinae, we have generated the most extensive molecular dataset to date for this subfamily. Additionally, we assembled a comprehensive dataset containing association data between coralliophiline snails and their cnidarian hosts. This involved the integration of literature data with collection data pertaining to the specimens used in our analyses. Moreover, DNA barcoding of the stomach content of the snails, or of the coral host sample, when collected with the molluscs, has been incorporated in our dataset.

In this study, phylogeny and association data were integrated with data on the depth range of the examined coralliophiline species. By mapping trophic ecology and habitat data on the phylogenetic hypothesis, we carried out macroevolutionary analyses aiming to:

1. Reconstruct the evolution of the trophic ecology in Coralliophilinae, by identifying the ancestral host for the subfamily and the occurrence of host shifts across the phylogenetic tree
2. Test the hypothesis that the Coralliophilinae originated in shallow waters, with subsequent repeated colonisations of deep habitats
3. Investigate the occurrence of clade-specific rate shifts across the Coralliophilinae radiation, which might be suggestive of a diversification driven by the acquisition of key adaptive innovations.

## Material and methods

### Material examined

**Fig. 2:**
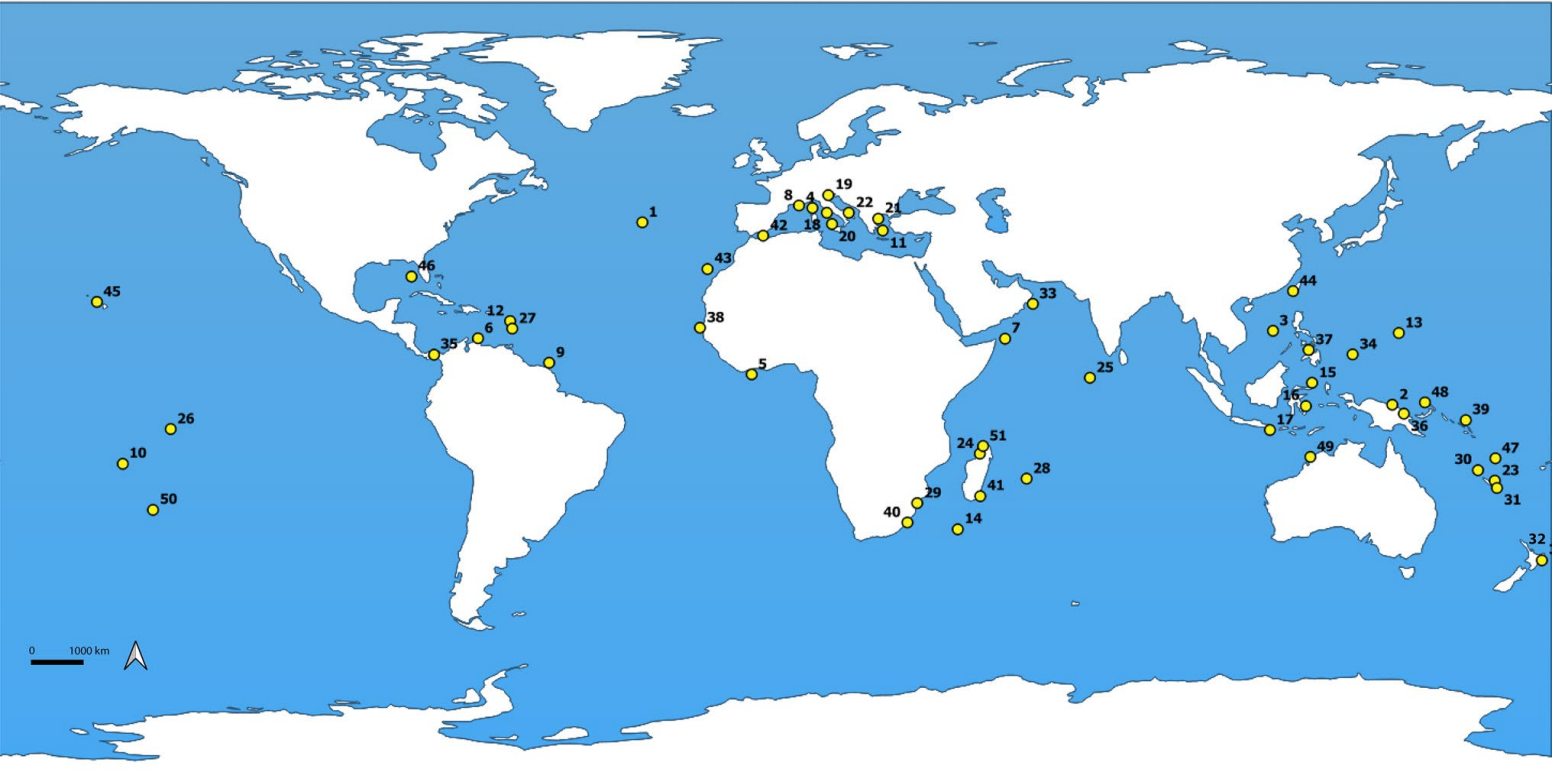
Global map featuring sampling locations of the Coralliophilinae dataset created using QGIS. See Table S1 for corresponding locality identifiers

The dataset consisted of 586 specimens (plus five for the out-group), morphologically ascribed to 111 species belonging to 10 of the 13 accepted extant genera of Coralliophilinae (WoRMS 2023). Specimens from tropical, subtropical and temperate areas were included (Fig. 2). A total of 74 specimens were collected by one of the authors (M.O.) or kindly provided by Francisco Otero of Las Palmas de Gran Canaria University. Two samples were provided by the Florida Museum (UF), one by the Museum of New Zealand (NMZN) and one by KwaZulu-Natal Museum (NMSA). The majority of samples (350) belonged to the Muséum national d’Histoire Naturelle (MNHN), Paris (catalogue numbers MNHN-IM), having been collected during several scientific expeditions (www.expeditions.mnhn.fr). Sequences of 163 additional specimens were obtained from GenBank.

Samples were collected from 0 m to approximately 1000 m depth and fixed in the field specifically for molecular analyses. The majority of shells were kept intact for identification and deposited as vouchers in the collections of MNHN, NMZN, UF, NMSA and the Department of Biology and Biotechnologies ‘Charles Darwin’ (BAU). In some cases, coralliophiline samples have been collected with their host and stored together. When the host was missing and the coralliophiline whole body was available, the stomach and foregut were dissected and processed for the amplification of cnidarian DNA directly from the gut content (Oliverio et al. 2009a) using cnidarian primer sets. Sequences from five Muricidae species were used as out-groups: *Orania rosea* (Houart, 1996)*; Pascula darrosensis* (Smith, 1884); *Claremontiella nodulosa* (Adams, 1845); *Semiricinula squamosa* (Pease, 1868); *Mancinella herberti* (Houart, 1998). For details on samples and GenBank accession numbers, see the Supporting Information (Table S1).

### Molecular analyses and gastropod species delimitation

Laboratory work was carried out in part at the Service de Systématique Moléculaire (UAR 2AD, MNHN, Paris, France) and in part at Molecular Systematic Laboratory, Department of Biology and Biotechnologies ‘Charles Darwin’, Sapienza University of Rome, Italy. For samples processed at MNHN, total genomic DNA was extracted from the foot and, when available, also from the stomach, using the Macherey–Nagel NucleoSpin 96 Tissue Kit and following the manufacturer’s protocol. For samples processed at Sapienza University of Rome, total genomic DNA was extracted using ‘salting out’ protocols (Fassio et al. 2023). Cnidarians DNA extraction was performed following Nocella et al. (2024). Polymerase chain reactions were performed following Fassio et al. (2022). In both laboratories, the same primers and amplification protocols were used. For molecular analysis and gastropod species delimitation details, see Supplementary Part 1.

### Cnidarian host identification and depth range

Cnidarians samples collected with the associated coralliophilines (for a few of which field observation and/or photographs were also available) were observed under a Wild-M6 microscope. Our examination focused on the arrangement and distribution of polyps and/or the calcareous skeleton. Taxa were identified following Fabricius and Alderslade (2001), Zibrowius (1980), and Veron (2000). To gather anatomical data, alcohol-preserved specimens were observed and dissected under a Wild-M6 microscope.

All *16S* and *ITS2* cnidarian nucleotide sequences, either obtained from the cnidarian samples associated with the snails or from the stomach contents, were matched with sequences available in Genbank with the NCBI BLAST web interface (https://blast.ncbi.nlm.nih.gov/Blast.cgi). Molecular taxonomic attributions were preliminarily determined using the BLAST taxonomy lineage report, taking in consideration all IDs with per cent of identity ≥ 89% and e-value ≤ 4e−04. Preliminary taxonomic identification was then cross-referenced for consistency with morphology (when the host sample was available) and geographical range. Cnidarian identification was conducted to the lowest taxonomic level possible and in all cases at least to the family level.

Recognising the considerable challenges in morphologically identifying coralliophiline and cnidarian species, literature records of coralliophiline-coral associations were considered reliable only when supported by morphological (on actual specimens or field photographs) or genetic identification pertaining to both gastropods and cnidarians.

The habitat of each species (deep v. shallow) was determined based on literature data (e.g. Oliverio 2009; Marshall and Oliverio 2009), on collection data of the assayed specimens, and on M.O. pers. observations. Species have been assigned to a shallow habitat (“S”, in Table S1) if living specimens are consistently collected in the euphotic zone (including cases with occasional collections of living specimens in the upper mesophotic, or empty shells from deeper habitats). Species have been assigned to a deep habitat (“D”, in Table S1) if living specimens are collected in the mesophotic or aphotic zone.

### Phylogenetic reconstruction and temporal calibration

Phylogenetic analyses were performed on the three single-gene datasets (*cox1* partitioned by codon, *16S* and *ITS2*) and on the combined dataset (*cox1* partitioned by codon + *16S* + *ITS2*) with both maximum likelihood (ML) and Bayesian inference (BI). For the combined dataset, only the specimens with at least the *cox1* data were kept. Sequences alignment, maximum likelihood and Bayesian analyses were performed following Nocella et al. (2024).

We identified four reliable fossil records of Coralliophilinae in the literature suitable for our phylogeny (Table 1). The first appearance of this neogastropod lineage is reported for the Middle Eocene of Mississippi and Louisiana in the Claiborne formation (42–44 mya; Dockery 1986) with the earliest known species attributed to the subfamily, *Coralliophila (Timothia) aldrichi* (Cossmann, 1903). The oldest known record of the genus *Galeropsis* Hupé, 1860, is from the Aquitanian, Early Miocene (20.44–23.03 mya) with *Galeropsis lavenayanus* Hupé, 1860 (Lozouet and Renard 1988). *Coralliophila richardi* P. Fischer, 1882, and *Hirtomurex squamosus* Bivona & Bernardi, 1838, are witnessed by the early Pleistocene in the Mediterranean Sea (0.77–2.58 mya; Vazzana 1996).

**Table 1:** Oldest fossil records for coralliophilines

Node	Oldest fossil records	Used calibration point mya	References	BI results 95% HPD
Coralliophilinae	Middle Eocene	42–44	Dockery (1986)	41.65–43.03
*Galeropsis*	Early Miocene	20.44–23.03	Lozouet and Renard (1988)	18.53–23.91
*Coralliophila richardi*	Early Pleistocene	0.77–2.58	Vazzana (1996)	0.77–2.39
*Hirtomurex squamosus*	Early Pleistocene	0.77–2.58	Vazzana (1996)	0.77–2.77

The four time-ranges used as calibration points; date intervals (95% HPD) obtained from BEAST (BI) analyses for selected major nodes

We selected a single specimen (indicated by an asterisk in Table S1) for each SSH retrieved with the integrative approach, to create a new combined dataset comprising 128 samples (including the out-group). Temporal calibration analysis was performed following Nocella et al. (2024).

### Diversification rates through time and ancestral state analysis

Macroevolutionary dynamics of diversification and ancestral state reconstruction were modelled across the phylogeny using the Bayesian analysis of macroevolutionary mixtures (BAMM v.2.5.0: Rabosky 2014) on the maximum clade credibility tree obtained in BEAST, after out-group removal, following Nocella et al. (2024). We reconstructed the ancestral states for the depth range, using Deep v. Shallow as character states; either the families or the orders (Scleractinia, Actiniaria, Malacalcyonacea, Antipatharia and Zoantharia) of cnidarian hosts exploited by each coralliophiline species were used as character in two distinct analyses of the trophic ecology. Ancestral state reconstructions were carried out using the Bayesian binary Markov chain Monte Carlo (BBM; Ali et al., 2012) in Reconstruct Ancestral State in Phylogenies (RASP, v. 4.2: Yu et al. 2015) Analyses priors were set following Nocella et al. (2024).

## Results

### Integrative taxonomy

The molecular dataset included 972 gastropod sequences (542 *cox1*, 344 *16S*, 87 *ITS2*) from 586 in-group and 5 out-group specimens. The combined dataset included sequences from 542 samples (after the removal of samples for which no *cox1* sequences were available). Preliminary identification of the 586 in-group specimens retrieved 116 PSH (primary species hypothesis). We excluded the amplification of nuclear pseudogene of the *cox1*, by checking the absence of indels and of internal stop codons, and the vast prevalence of 3rd position substitutions. All the 481–658 bp long *cox1* sequences (516, thus excluding 21 shorter minibarcodes) were included in the ASAP analysis, which divided the dataset into 67–118 hypothetical species (in the ten best partitions: Supporting Information, Fig. S1).

The first and second ASAP best partitions (threshold distance = 11, 12%, number of species 67, 71, respectively) evidently overlumped multiple clearly distinct entities*.* For instance, these partitions merged into a single species hypothesis samples showing substantial differences in shell morphology (i.e. the smooth-shelled *C. violacea, C. galea, C. erosa, C. salebrosa, C. radula,* almost all *Leptoconchus* spp.*,* along with the spiny-shelled *H. squamosus* A and B*, B. mansfieldi, B. bernardi, H. filiaregis* A), making a largely polyphyletic assemblage. The same holds for the seventh and ninth partitions, which were not considered due to their high threshold distance (10%) again resulting in inconsistent overlumping. The partitions 4–5, 6, 8, and 10 in the rank (threshold distance = 4–5.5%; number of species 104–117) were only slightly less splitting than partition 3; the latter (threshold distance = 3.9%; number of species 118) mostly aligned with our shell morphology results, was not contradicted by the phylogenetic ones, and was thus retained as the most reliable.

All the hypothetical species, except four, identified by ASAP in the third-best partition corresponded to monophyletic groups highly supported in the ML and BI phylogenetic reconstructions: Ufb (Ultrafast bootstrap) = 99–100, PP (Posterior Probability) = 0.98–1. The exceptions were *Hirtomurex oyamai* (Ufb = 75, PP = 1) not supported in the ML combined tree, and *Mipus vicdani* (Ufb = 100, PP = 0.86) in the BI, whereas *Coralliophila fearnleyi* and *Coralliophila violacea* A were not supported in any of the combined trees.

Almost all specimens that were not included in the ASAP analysis because their *cox1* sequence was too short, did not represent new independent lineages in the phylogenetic analyses, but ended up into clades corresponding to hypothetical species already identified by ASAP, except five: MNHN-IM-2009-5423 *Babelomurex nagahorii*, MNHN-IM-2013-11444 *Leptoconchus* sp. H, MNHN-IM-2013-12027 *Coralliophila bulbiformis*, BAU-2892 *Babelomurex benoiti*, BAU-2968 *Coralliophila basileus*. These five samples formed five lineages clearly distinct from all other clades in the combined tree and we consider that they represent five additional hypothetical species.

In four cases, two or more PSH were retrieved in all the ASAP partitions as a single hypothetical species (confirmed by the phylogenetic analyses): (1) *Leptoconchus ingranulosa, L. incycloseris, L. inpleuractis, L. ingravis* (Ufb = 100, PP = 0.99). Among the species identified by Gittenberger and Gittenberger (2011), *L. inpleuractis* and *L. ingravis* were not retrieved as reciprocally monophyletic, but rather intertwined within a single clade, which also includes a sequence (MNHN-IM-2009-6385 *Leptoconchus* sp.) generated in this study. Conversely, the sequences assigned to *L. ingranulosa* and *L. incycloseris* were reciprocally monophyletic. However, the genetic distance calculated among the four morphospecies ranged 1.2–3.8%. The inter-/intraspecific threshold corresponding to the partition chosen in this work is 3.9%, which resulted in consolidating this clade of four morphospecies into a single SSH (secondary species hypothesis). (2) *Leptoconchus inpileus* and *L. infungites* (Ufb = 100, PP = 1). These sequences were reciprocally monophyletic; however, their rather low genetic distance (1.6%) is suggestive of another case of synonymy; (3) *Babelomurex cariniferoides* and *Latiaxis nippoleifera* (Ufb = 100, PP = 1). The single specimen of *B. cariniferoides* was collected in New Caledonia, in the same locality of one specimen ascribed to *L. nippoleifera.* (4) *Coralliophila richardi* and *Emozamia licina* (Ufb = 100, PP = 1). The specimens ascribed to these two nominal species, from the Caribbean (Harasewych et al. 2022), New Zealand and Mediterranean (Russini et al. 2023) were shown to represent a single genetic species, seemingly cosmopolitan.

In ten cases, one PSH was split by ASAP into two or more hypothetical species (each confirmed as monophyletic by the phylogenetic analyses): (1) *Galeropsis monodontus* into eight SSH (Ufb = 99–100, PP = 0.99–1). This is a remarkable case of cryptic diversity within Coralliophilinae. Our 27 samples morphologically ascribed to this nominal species, actually belong to eight different molecular species. Samples of different SSH displayed no differences in shell shape, were frequently collected in the same locality and occasionally from the same station, and we retrieved no evidence of host differences; (2) *Coralliophila fimbriata* into five SSH (Ufb = 100, PP = 1). It emerged as the second most splitted nominal species in our dataset. Samples collected in the same locality belonged to different SSH; (3) *Coralliophila pulchella* into two SSH (Ufb = 97–100, PP = 1). Samples are found to belong to two distinct sister species, one corresponding to samples collected in the Philippines, South Madagascar and Papua New Guinea, and the other from the Maldives; (4) *Rapa rapa* into three SSH (Ufb = 100, PP = 1). The three specimens ascribed to this coralliophiline nominal species, actually correspond to three distinct species. *R. rapa* B and C were collected in the same locality; (5) *Hirtomurex filiaregis* into four SSH (Ufb = 100, PP = 1). The four samples morphologically ascribed to this morphospecies actually correspond to as many different species. Notably, *H. filiaregis* A is phylogenetically quite unrelated to all the other species in all phylogenetic trees, despite having been collected in the same area (New Caledonia) as *H. filiaregis* C; (6) *Coralliophila violacea* (the type species of the genus *Coralliophila*) into three SSH (A not supported, B represented by a single samples and C Ufb = 99, PP = 0.97). As already noted by Simmonds et al. (2018), it actually represents a complex of cryptic species. Of the three putative species detected herein, *C. violacea* B is represented by only one sample, and *C. violacea* A lacks consistent phylogenetic support in the combined trees. Consequently, the species delimitation for this complex remains uncertain; (7) *Babelomurex japonicus* into two SSH (Ufb = 100, PP = 1). These two clades (the first comprising one sample collected in the China Sea and the second comprising four samples from the New Caledonia) do not emerge as sister species in any combined trees; (8) *Babelomurex spinosus* into two SSH (Ufb = 100, PP = 1). These two specimens (collected one in the China Sea and one in New Caledonia) correspond to two distinct sister species; (9) *Coralliophila costularis* into two SSH (Ufb = 100, PP = 1). These samples are identified as two sister species one corresponding to samples collected in Papua New Guinea and the other from various eastern Indian Ocean localities (Madagascar, Mozambique and Yemen); (10) *Hirtomurex squamosus* into two SSH (Ufb = 100, PP = 1). The *cox1* sequence of *H. squamosus* A is shorter than those representing the putative species B, which may have biased the ASAP analysis. However, considering that the specimens of the two hypothetical species feed on different cnidarian families, we have preferred to keep them into two separate SSH, which has not affected the subsequent analyses.

**Fig. 3:**
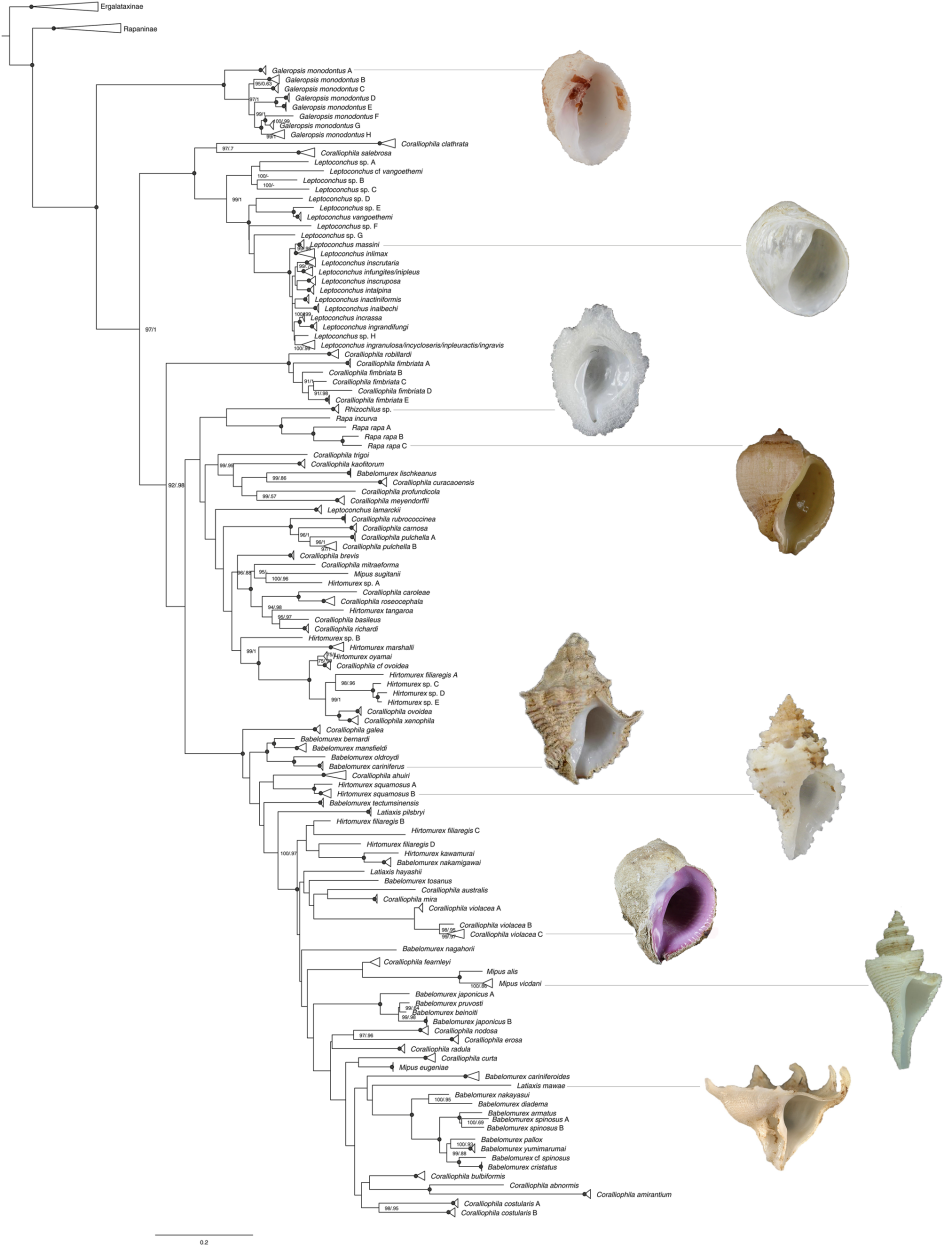
Phylogenetic relationships of the subfamily Coralliophilinae (maximum likelihood tree on combined dataset), with clades collapsed by species. Numbers at nodes indicate branch support values [ultrafast bootstrap (Ufb) values and posterior probability (PP), respectively]; support values are shown only when at least one of them is ≥ 95%; black circles at nodes indicate maximum support (Ufb = 100, PP = 1). Photograph credits: Mélanie Van Weddingen (MNHN)

In the integrative taxonomic process, we identified a total of 123 SSH as depicted in the collapsed tree (Fig. 3). The PSHs that have undergone further subdivision into multiple SSHs were named with alphabetical suffixes from A to H.

### Phylogenetic reconstruction

The final alignment comprises 658 bp of *cox1*, 469 of *16S* and 785 of *ITS2*. Comparison of the major nodes among the ML and BI combined trees revealed no inconsistencies (Supporting Information, Figs S2–S9): the monophyly of the subfamily Coralliophilinae was strongly supported (Ufb = 100, PP = 1), while all the genera as traditionally conceived, except *Galeropsis* and *Rapa*, did not prove monophyletic.

*Leptoconchus* was split into two lineages, one corresponding to *Leptoconchus lamarckii* (Ufb = 100, PP = 1) (the type of *Magilopsis* G. B. Sowerby III, 1919), and one including all the other *Leptoconchus* species (Ufb = 99, PP = 1).

The three species traditionally ascribed to the genus *Latiaxis* (the type species *Latiaxis mawae, L. pilsbryi,* and *L. hayashii*) represented three distinct lineages.

The species of the genus *Mipus* were split into three lineages: two corresponded respectively to *M. sugitani* (Ufb = 100, PP = 1) and *M. eugeniae* (Ufb = 100, PP = 1), the latter included *M. vicdani* and *M. alis* (Ufb = 100, PP = 1).

The species morphologically ascribed to the genus *Hirtomurex* were split into ten lineages, with a high number of cryptic species: (i) *Hirtomurex* sp. A, (ii) *H. tangaroa*, (iii) *Hirtomurex* sp. B, (iv) *H*. *marshalli,* (v) *H. oyamai,* (vi) *H. filiaregis* A*;* (vii) *H. kawamurai* (Ufb = 100, PP = 1); (viii) *H. filiaregis * B and *Hirtomurex* sp. C-D-E (Ufb = 98, PP = 0.96); similarly, (ix) *H. filiaregis* C and D (Ufb = 100, PP = 1); (x) the type species *H. squamosus* that was split into two cryptic species, *H. squamosus* A and B (Ufb = 100, PP = 1).

The genus *Babelomurex* included 9 lineages: (i) *B. lischkeanus* (Ufb = 100, PP = 1); (ii) *B. bernardi, B. mansfieldi, B. oldroydi* and the type species *B. cariniferus* (Ufb = 100, PP = 1); (iii) *B. tectumsinensis* (Ufb = 100, PP = 1); (iv) *B. nakamigawai* (Ufb = 100, PP = 1); (v) *B. tosanus* (Ufb = 100, PP = 1); (vi) *B. nagahorii*; (vii) *B. pruvosti, B. benoiti* and *B. japonicus* A and B (Ufb = 100, PP = 1); (viii) *B. cariniferoides* (Ufb = 100, PP = 1); (ix) *B. nakayasui, B. diadema, B. armatus, B. pallox, B. yumimarumai, B.* cf *spinosus, B. cristatus* and *B. spinosus* A and B (not supported).

The species traditionally ascribed to the genus *Coralliophila* included twenty lineages. *C. trigoi, C. kaofitorum, C.* c*uracaoensis, C. brevis, C. mitraeforma, C.* cf *ovoidea, C. galea, C. ahuiri, C. fearnley* and *C. curta* represented 10 distinct lineages The remaining 10 clades included: (1) *C. clathrata*, sister to *C. salebrosa* (Ufb = 100, PP = 1); (2) *C. robillardi* and the five species identified as *C. fimbriata* A-E (Ufb = 100, PP = 1); (3) *C. profundicola*, sister to *C. meyendorffii* (Ufb = 100, PP = 1); (4) *C. rubrococcinea, C. carnosa* and the two species identified as *C. pulchella* A and B (Ufb = 100, PP = 1); (5) *C. caroleae*, sister to *C. roseocephala* (Ufb = 100, PP = 1); (6) *C. basileus*, sister to *C. richardi* (Ufb = 95, PP = 0.97); (7) *C. ovoidea*, sister to *C. xenophila* (Ufb = 100, PP = 1); (8) *C. australis, C. mira* and the type species *C. violacea* splitted into three species A-C (not supported); (9) *C. nodosa, C. radula* and *C. erosa* (not supported); (10) *C. bulbiformis, C. amirantium* and the two species identified as *C. costularis* A and B (not supported).

Hereafter we will use *Leptoconchus* to indicate the clade including all *Leptoconchus* species except *L. lamarckii*, and *Babelomurex* sensu stricto for the clade comprising *B. bernardi, B. mansfieldi, B. oldroydi* and the type species* B. cariniferus.*

### Dating major lineages

**Fig. 4:**
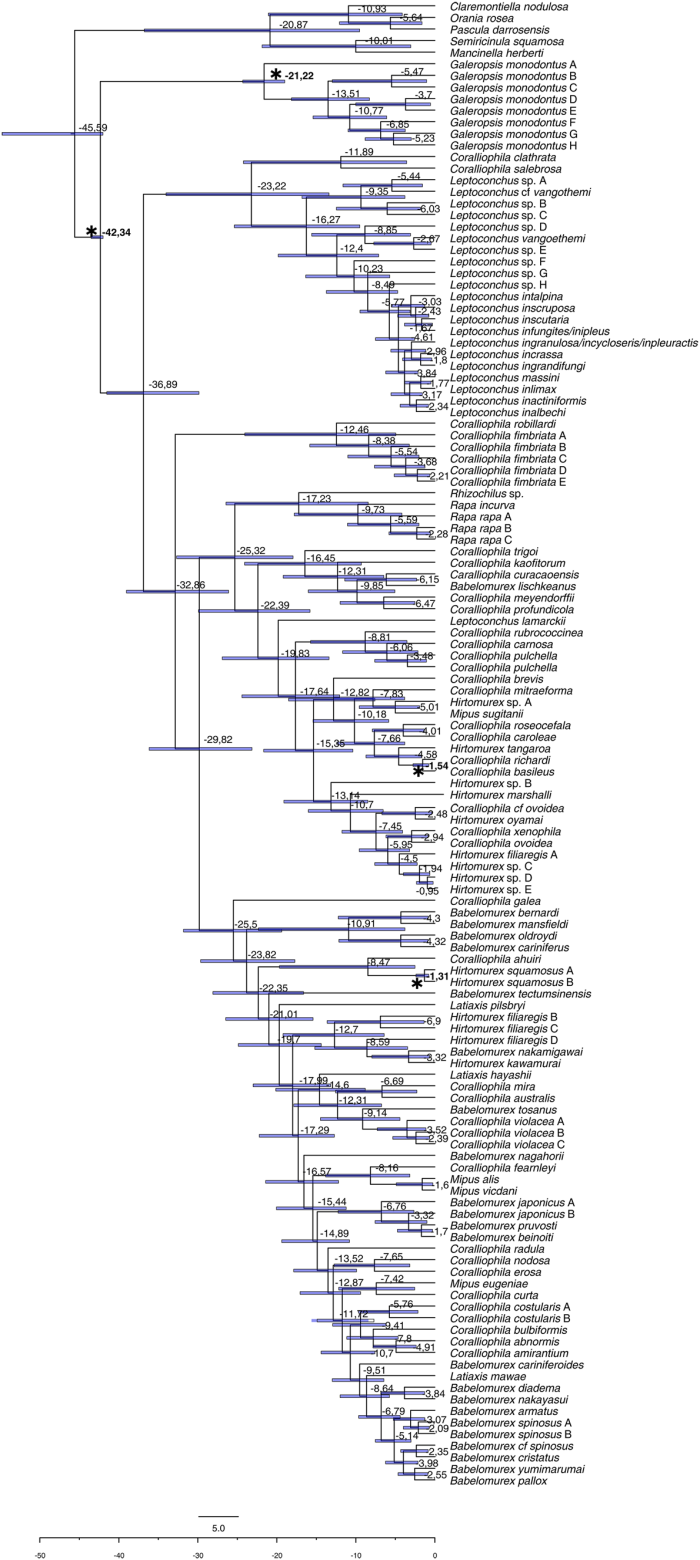
Time calibrated, single species phylogenetic reconstruction obtained using BEAST on combined dataset. Bars at nodes indicate 95% confidence intervals of ages, expressed in mya. Asterisks (and numbers in bold) indicate the nodes (and the relevant fossil-based datings) used to time-calibrate the tree

All our calibration nodes were dated in the BEAST output congruently with the corresponding fossil data. The time-calibrated phylogeny (Fig. 4; see Table 1 for 95% HPD) estimated the origin of the subfamily Coralliophilinae at 42.34 mya (95% HPD 41.65–43.03) during the Lutetian (Middle Eocene). The origin of the genus *Galeropsis* was dated at 21.22 mya (95% HPD 18.53–23.91) from the Burdigalian to the Aquitanian (Early Miocene). The *Hirtomurex squamosus* complex was estimated as having originated 1.3 mya (95% HPD 0.77–2.39) during the Meghalayan (Holocene). Similarly, *Coralliophila richardi* diverged from *C. basileus* approximately 1.54 mya (95% HPD 0.77–2.77). The origin of *Leptoconchus* was dated at 12.4 mya (95% HPD 9.5–25.37) during the Miocene. *Babelomurex* sensu stricto was estimated as having originated during the Miocene, with the node dated at 10.9 mya (95% HPD 3.78–22.32). *Rapa* was estimated to have originated around 9.73 mya (95% HPD 4.18–17.79) in the Late Miocene. The clade including *C. rubrococcinea, C. carnosa, C. pulchella* A and B*, C. brevis, C. mitraeforma, Hirtomurex* sp*.* A*, M. sugitanii, C. roseocephala, C. caroleae, H. tangaroa, C. richardi, C. basileus, Hirtomurex* sp*.* B*, H. marshalli, C.* cf *ovoidea, H. oyamai, C. xenophila, C. ovoidea, H. filiaregis* A*, Hirtomurex* sp*.* C-D-E*,* was estimated as having originated 17.64 mya (95% HPD 12.04–24.42) during the Late Miocene.

### Diversification rate of Coralliophilinae

**Fig. 5:**
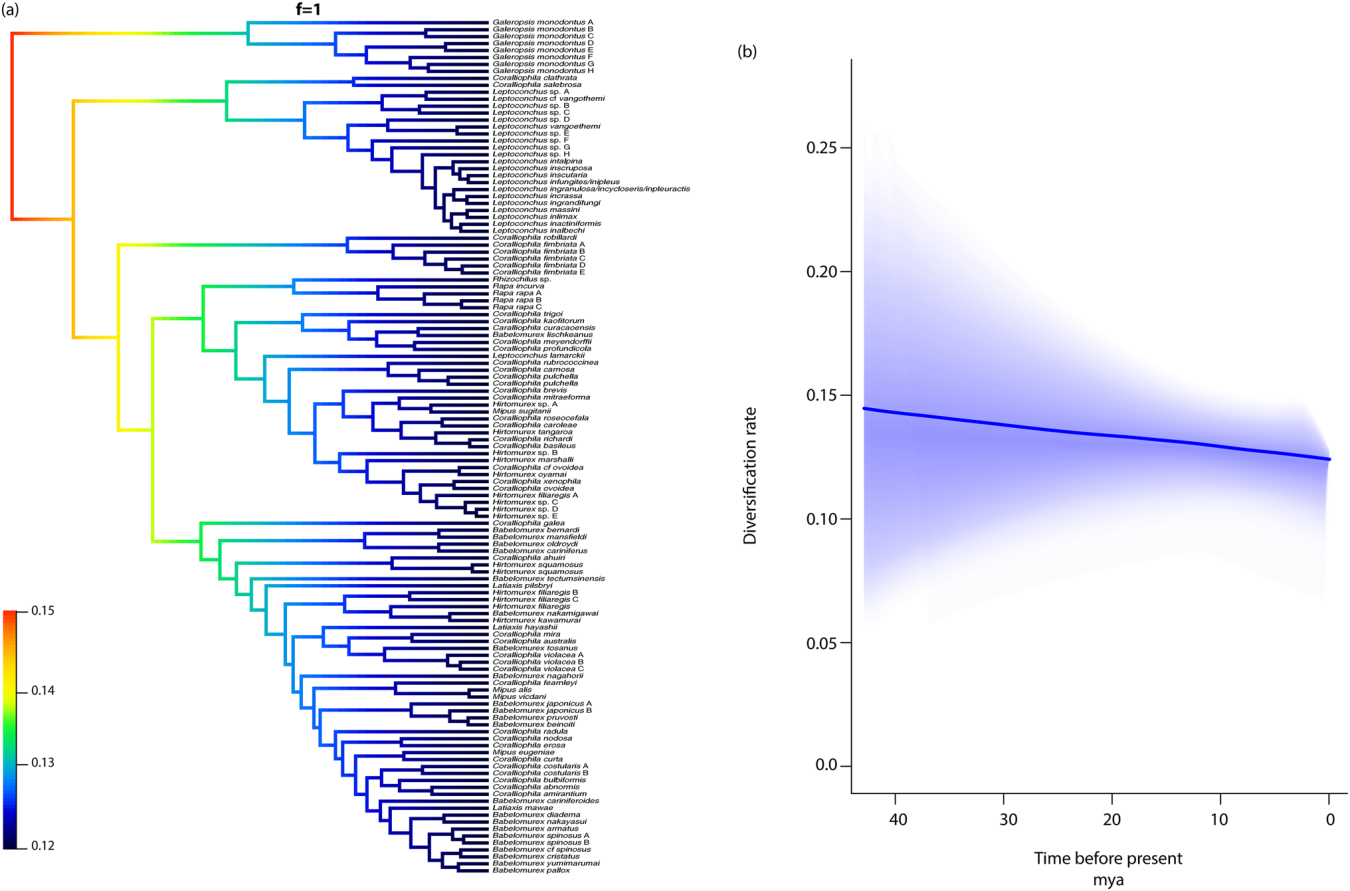
Diversification rates variation within Coralliophilinae across clades and time. **a** The single BAMM credible shifts plot representing the rate shift configuration and a posterior probability shift configuration corresponding to 1. **b** BAMM plot depicting the net diversification rates through time

We modelled the diversification rates within the Coralliophilinae as a function of time. The best model supported by BAMM analysis indicated a steady rate of diversification over time, with a posterior probability of 1. The analysis consistently upheld this model, demonstrating alignment with every significant shift in the rate of speciation considered in our prior assessments. The credible shifts plot depicted a non-core shift across all lineages (Fig. 5a), with a 100% posterior probability. The rate-through-time BAMM plot supported a scenario with an initial higher rate of diversification (speciation rate ∼0.15) that decreased gradually over time (to ∼ 0.12) until the present day (Fig. 5b).

### Ancestral state reconstruction

**Fig. 6:**
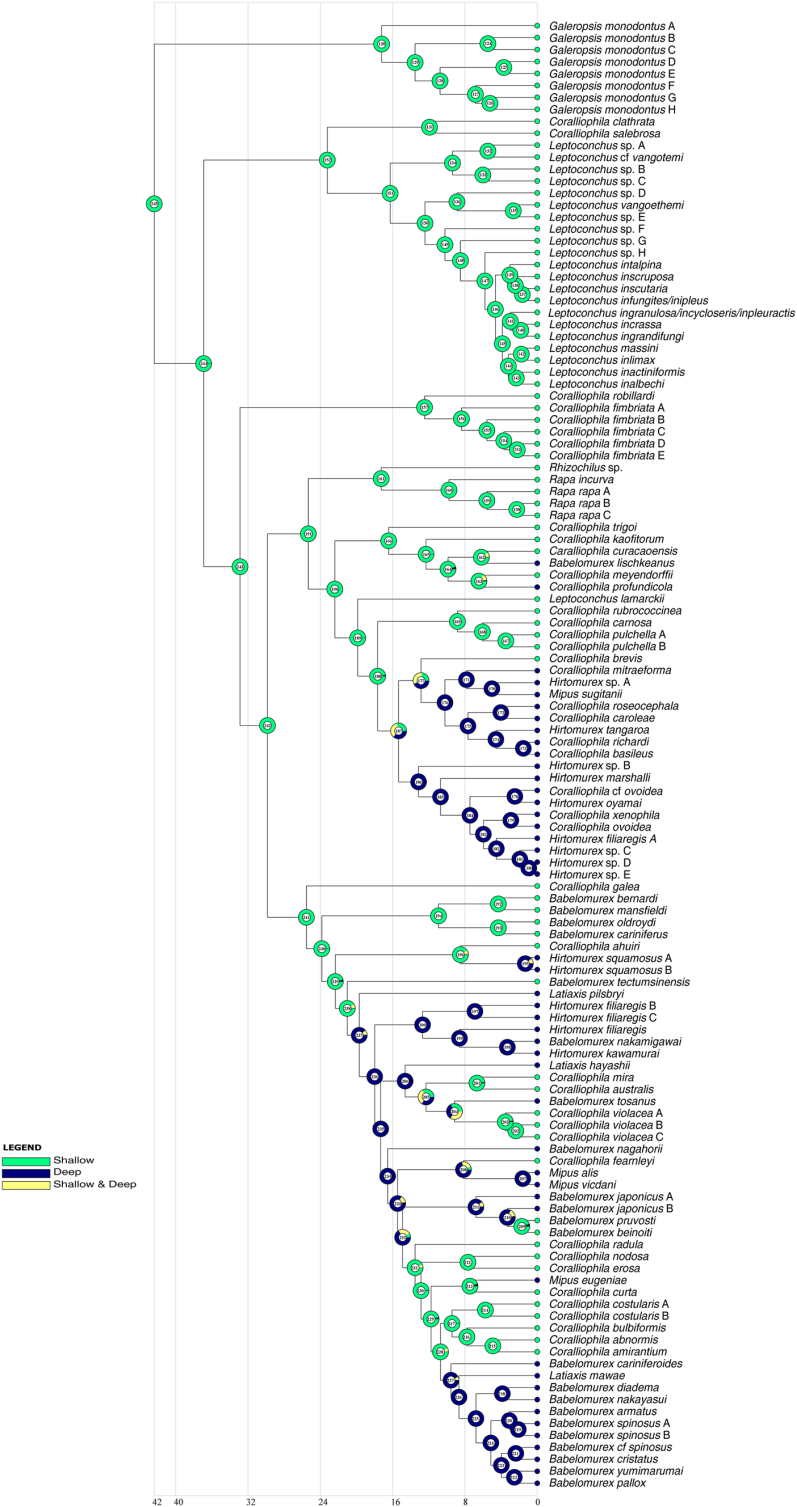
Graphical representation of the ancestral state reconstruction at each node of the phylogeny of the subfamily Coralliophilinae obtained from RASP by BBM analysis using depth range as a prior. Pie charts at each node (from 124 to 222) show the probabilities of alternative ancestral states; numbers inside the pie charts identify each node. The legend shows the colour key to the depth range. X-axis represents time in millions of years

Of the 123 species recognised by our integrative taxonomy approach, 75 (61%) come from shallow-water habitats, while 48 (39%) inhabit deep waters. The results of the ancestral state reconstruction for habitat types (shallow vs. deep) are shown in Fig. 6. (Nodes are numbered to ease following the description). The ancestral Coralliophilinae (node 245) were estimated to have evolved in shallow waters (99.9% marginal probability), which were retrieved as the ancestral habitat for most clades. The first shift to deep waters took place at node 237 (89.5% deep), followed by a reversal to shallow water at node 231 (93.6%) that gave rise to several common shallow-water species (e.g. *C. radula, C. erosa, C. bulbiformis*). Finally, another shift to deep waters is estimated to have occurred at node 227 (93%), subtending one of the spiny clades. A second shift to deep waters likely occurred at node 187 (37% deep, 35% both shallow and deep), in the ancestor of 19 species of which only *Coralliophila brevis* is estimated to have secondarily migrated back into shallow waters. Several additional shifts to deep waters were retrieved scattered across the tree: *Babelomurex lischkeanus, Coralliophila profundicola*, the pair *Hirtomurex squamosus* A–B, and *Mipus eugeniae*. Instances of reversals to shallow waters were also observed: the pair *C. mira*—*C. australis*, *C. violacea* A–C, *C. fearnleyi*, and the pair *B. pruvosti*—*B. benoiti*.

A total of 82 distinct coralliophiline-cnidarian associations were identified (Table S3). We have identified the cnidarian hosts (26 families) of 51% (63) of the tested gastropod species. For 36 coralliophiline species the host was retrieved exclusively from literature, whereas for the remaining 46 host identification was based on our molecular and/or morphological analyses. Among them, 15 resulted from DNA barcoding of coralliophiline stomach content (17 sequences, out of the 72 stomach samples), 6 from coral tissue amplification, and 25 were exclusively identified by morphology due to the unavailability of coral tissue or unsuccessful DNA amplification. When multiple association records were available for a species (in 26 instances), usually they congruently indicated its association with a same cnidarian family, with six exceptions (*B. tectumsinensis, B. cariniferus, C. meyendorffii, C. richardi, C. salebrosa* and *C. galea*). Congruence was even higher in analysing the association with cnidarian orders, with only two exceptions (*Coralliophila salebrosa* and *C. meyendorffii*).

**Fig. 7:**
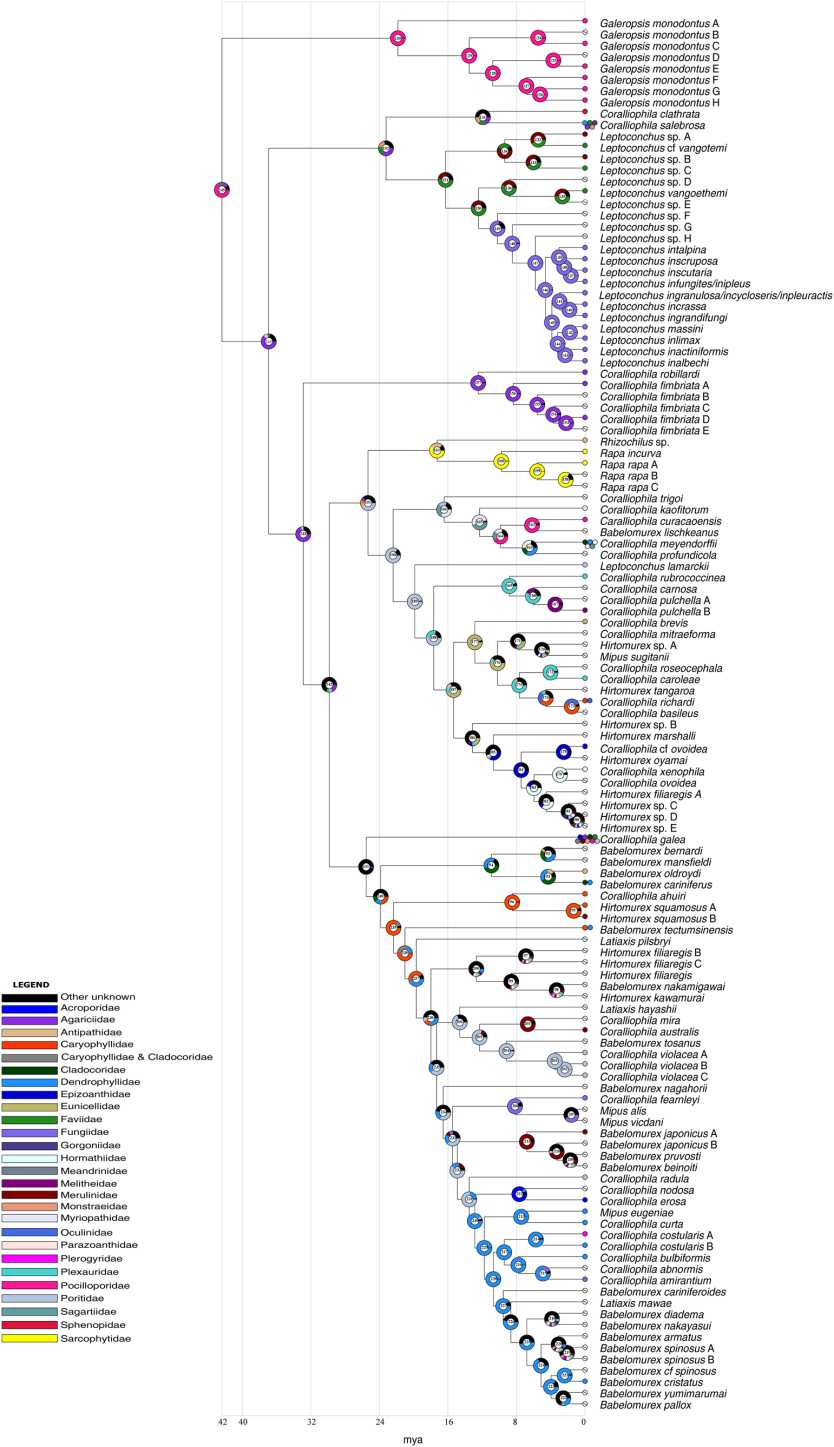
Graphical representation of the ancestral state reconstruction at each node of the phylogeny of the subfamily Coralliophilinae obtained from RASP by BBM analysis using cnidarian families as a prior. Pie charts at each node (from 124 to 222) show the probabilities of alternative ancestral states; numbers inside the pie charts identify each node. The legend shows the colour key to the hosts; black represents other unknown ancestral states. White barred circles represent no host information. X-axis represents time in millions of years

The results of the ancestral state reconstruction using cnidarian families as prior for the states (Fig. 7) show that for the Coralliophilinae (node 245) the scleractinian family Pocilloporidae was the most likely inferred ancestral host (65.8% marginal probability), followed by a multiple association with Caryophyllidae and Cladocoridae (13%) and by Agariciidae (11.1%). Pocilloporidae resulted as the most likely ancestral host also for the genus *Galeropsis* (node 130, 98.9%). At node 151, Faviidae was estimated as the ancestral host of the clade comprising all the species belonging to the genus *Leptoconchus* (52.7%), followed by Merulinidae (26%). At node 160 Sarcophytidae was the most likely ancestral host of the genus *Rapa* (99%). Sarcophytidae resulted as the ancestral host also for a clade including *Rapa* and *Rhizochilus* (node 161, 61.2%). The gastropod-coral associations among other coralliophiline major clades did not display a phylogenetically consistent pattern: different species within the same clade in our tree feed frequently on different cnidarian families. However, the clade comprising *Coralliophila robillardi* and the five species identified as *C. fimbriata* A-E (node 157), exhibited a clear pattern, with the ancestor of this clade estimated to have had Agariciidae as the most likely host (97.7%).

**Fig. 8:**
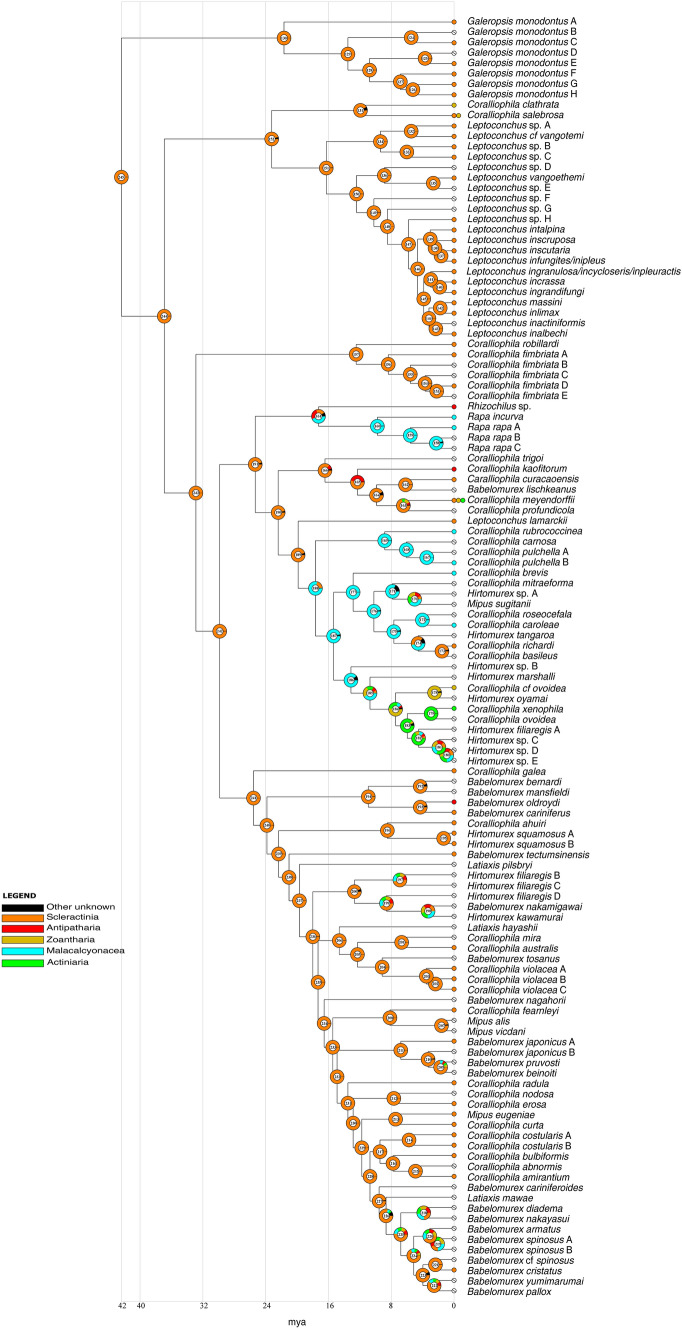
Graphical representation of ancestral state reconstruction at each node of the phylogeny of the subfamily Coralliophilinae obtained from RASP by BBM analysis cnidarian orders as a prior. Pie charts at each node (from 124 to 222) show the probabilities of alternative ancestral states; numbers inside the pie charts identify each node. The legend shows the colour key to the hosts; black represents other unknown ancestral states. White barred circles represent no host information. X-axis represents time in millions of years

In the ancestral state reconstruction using cnidarian orders as prior for the states (Fig. 8), the ancestral Coralliophilinae (node 245) were estimated to be associated with scleractinian Hexacorallia (99.4% marginal probability). Scleractinia were retrieved as the ancestral host for most clades. The main shifts occurred in the ancestral host of the genera *Rhizochilus* and *Rapa* (node 161, shift to Octocorallia Malacalcyonacea 43.9%) similarly to the ancestor of the clade comprising 23 species (node 188, shift to Octocorallia Malacalcyonacea, 79%). Within the latter clade, three species feed on hexacorals (one on Scleractinia, one on Zoantharia, one on Actiniaria), while the host of the remaining species, when known, is an Octocorallia Malacalcyonacea.

## Discussion

### The assessment of Coralliophilinae diversity

The integrative taxonomy analysis was imperative to correctly estimate the alpha-diversity of the Coralliophilinae and robustly infer the species tree, crucial for the subsequent macroevolutionary analyses.

We have significantly increased the taxonomic span of Coralliophilinae diversity, with molecular data for 46% of the accepted species and 77% of the accepted genera, compared to 12% of species and 54% of genera in previous works (Oliverio et al. 2009b).

The 116 morphospecies initially identified actually comprise 123 distinct species, with some exceptions discussed in detail below. Our analysis revealed only four instances of potential oversplitting of actual species diversity. Among them, particularly interesting is the case of *Leptoconchus ingranulosa/L. incycloseris/L. inpleuractis/L. ingravis,* since these four species were defined on the basis of their host preference, assuming a process of speciation driven by adaptation to the host (Gittenberger and Gittenberger 2011). While for *L. inpleuractis* and *L. ingravis* we are prone to consider the assayed specimens as quite evidently conspecific (low genetic distance, and non-reciprocally monophyletic), the two other nominal species (*L. ingranulosa* and *L. incycloseris*) may either represent two distinct species with a clear ecological distinction (being associated to different genera of Fungiidae), or a single species, distinct from *L. inpleuractis/L. ingravis*. Additional data would be needed to make a robust taxonomic decision.

Conversely, most instances of morphological-molecular incongruence indicated an underestimation of the actual species diversity, leading to the splitting of ten nominal species. This is a common situation in molluscs where cryptic species complexes are particularly abundant (e.g. Barco et al., 2008). Among them, particularly interesting for the high number of cryptic species detected, are the cases of *Galeropsis monodontus* and *Coralliophila fimbriata*, split into eight and five molecularly supported species, respectively.

Out of the 10 nominal extant genera represented in our dataset, a total of six (*Babelomurex, Coralliophila, Mipus, Leptoconchus, Latiaxis, Hirtomurex*) were not monophyletic as traditionally conceived. Although the present work is not aimed at reassessing the coralliophiline systematics, the following general taxonomic considerations may prove useful in a future work on the systematics of this subfamily.

*Galeropsis* proved monophyletic, comprising at least 8 cryptic species within the *G. monodontus* complex; it would be interesting to test whether *Purpura porphyroleuca* Crosse, 1870 (currently included in *Coralliophila* s.l.; Cernohorsky 1980: 114, figs. 4, 5) actually belongs in this genus as morphology suggests.

*Rapa* also proved monophyletic, with at least three cryptic species within *Rapa rapa*.

*Rhizochilus* was based on specimens of a single species, and both its relationships with *Rapa* (topological, but not supported by PP or Ufb), and its actual diversity need to be further tested.

The actual magnitude of the radiation of *Leptoconchus* needs to be re-evaluated; however, it proved monophyletic only if excluding *L. lamarcki*, for which *Magilopsis* G. B. Sowerby III, 1919 could be reinstated.

*Coralliophila robillardi* and the complex of *C. fimbriata* A-E may deserve a genus on their own, which might be the available *Coralliobia* H. Adams & A. Adams, 1853.

Five species are included in a clade that could be defined as *Babelomurex* s.s.: the type species *B. cariniferus*, along with *B. oldroydi, B. bayeri, B. mansfieldi, B. bernardi*. All the other species sharing a morphologically similar spiny shell are interspersed across the tree among smooth-shelled species: spiny and smooth shells have probably been acquired and lost multiple times during the coralliophiline evolution, implying that an increased number of species should be analysed before drawing any conclusions on the evolution of shell morphology in this subfamily.

The type species of *Coralliophila* (*C. violacea*, which is actually a complex of at least three cryptic species) is retrieved in our analyses as topologically related (albeit with a weak support) to *C. australis* and *C. mira*; however, the relationships with *C. bulbiformis*, *C. costularis* and *C. radula*, previously obtained in a molecular phylogenetic analysis of a reduced dataset (Oliverio et al. 2009b), has not been retrieved herein. While it is evident that the use of the genus *Coralliophila* (the type genus of the subfamily) should be restricted to a much smaller clade than currently done, the extent of this lineage needs to be better defined.

Overall, the phylogenetic patterns emerging from this work are more in contrast than in agreement with shell morphology-based traditional taxonomy, casting serious doubts on the validity of the current classification. The alternate hypothesis would weaken the reliability of molecular phylogenetic approaches based on mitochondrial genes. Indeed, the so-called ‘Davison-effect’, i.e. the accelerated accumulation of mutations in mitochondrial genes of protandrous hermaphrodites (Davison 2006), such as Coralliophilinae, may have obscured the phylogenetic signal of our dataset. However, preliminary data from a phylogenetic analysis on an Exon-Capture dataset (N.P. unpublished), which should be less affected by this issue (Abdelkrim et al. 2018), broadly confirmed the patterns retrieved herein, with *Galeropsis* as the most basal lineage among extant coralliophilines, *Leptoconchus* as sister to the remaining lineages, *Coralliophila* as strongly polyphyletic and the smooth-shelled *C. violacea* falling within a clade of spiny-shelled species. For this reason, we are more inclined towards considering shell morphology highly plastic probably in response to adaptive pressures, and therefore not suitable as an uncritical source of diagnostic features for supraspecific taxonomy, a pattern not unusual in gastropods (e.g. Fassio et al. 2021). Probably, only an in-depth reassessment of characters from the anatomy might provide morphological features suitable to delimit major lineages worthy of genus-level recognition in Coralliophilinae (Richter and Luque 2002).

### Macroevolutionary patterns

Robust molecular evidence indicates that the origin and early diversification of coralliophilines could have occurred between 41 and 43 mya (congruent with the age of the fossil *Coralliophila aldrichi*). Despite Coralliophilinae are known to be more diverse in deep water habitats, from the mesophotic zone down to bathyal and abyssal bottoms (75% of deep-water species: Oliverio 2008), our dataset is biased towards shallow-water taxa (61% of shallow-water species), as expected due to the difficulties in collecting in deep-water rocky bottoms. However, given the good taxonomic coverage obtained, we do not expect that this bias affects the results of the ancestral state reconstructions. Our analysis strongly supported the hypothesis that the Coralliophilinae originated in shallow waters with multiple subsequent instances of colonisations of deep waters (Oliverio et al. 2009b). At least three major shifts to deep habitats were detected (two shifts concern unsupported nodes, allowing, in alternative topologies, additional shifts to deep habitats).

Coralliophilinae feed on five cnidarian orders belonging to the anthozoan subclasses Hexacorallia and Octocorallia. The present study, that includes 41 new coralliophiline-cnidarian associations, demonstrates that the vast majority of coralliophiline species is rather specialised in its parasitic behaviour. Although, our dataset was based on a majority of single records per coralliophiline species (which may bias the perception towards a “one coralliophiline-one cnidarian” pattern), many of these specialised associations are confirmed by authors’ observation, reliable anecdotal reports or photographic material in scuba divers’ websites (e.g. http://www.www.poppe-images.com). In most cases, individuals of the same coralliophiline species feed on cnidarians belonging to a single family, with a few exceptions such as *C. galea*, which feeds on corals of ten different families, the broadest diet so far reported for the subfamily, and *C. meyendorffii* and *C. salebrosa*, each of them feeding on five families belonging to two different orders (see Table S3).

The BBM analysis indicated that shallow-water scleractinians were the most likely ancestral host for the Coralliophilinae. The Pocilloporidae served as host for the ancestral Coralliophilinae since the beginning of coralliophiline history (41–43 mya) and has been maintained until the present day in the genus *Galeropsis*. The association with scleractinians persisted in all major ancestral lineages except two. The first shift occurred 18–26 mya in the common ancestor of the genera *Rhizochilus* and *Rapa*, which is supposed to have moved to the octocoral Malacalyonacea; this association has persisted up to the present day in the genus *Rapa* (with Sarcophytidae), while the sister lineage *Rhizochilus* might have secondarily shifted back to hexacorals, evolving a new association with the family Anthipatidae. The second and major dietary shift occurred in the ancestor of the “node 188 clade”, which also moved to the octocoral Malacalcyonacea 18–20 mya. Within this clade, at least three reversals to hexacorals have occurred in deep waters: one to deep-water Scleractinia carried out by *C. richardi* (2–5 mya); another in *C.* cf *ovoidea* to Zoantharia (3–8 mya); the third in the ancestor of the clade comprising *C. xenophila, C. ovoidea, H. filiaregis A* and the three species of *Hirtomurex* (sp. A-C), that moved to Actiniaria approximately 6–7 mya. Among the remaining clades, some species are not associated with Scleractinia (*C. kaofitorum* and *B. oldroydi*), or are clearly polyphagous (*C. meyendorffii*), but these instances did not bear any influence on the ancestral nodes’ host probability. Both shifts to octocorals actually concern nodes with weak or no support in our phylogeny (Fig. 3); alternative topologies may require more shifts, and we have discussed only the most conservative scenario.

We did not retrieve a consistent pattern for the major coralliophiline clades, some of which display a clear trend of specialisation, whereas others exhibit a higher degree of dietary variance. The *Galeropsis monodontus* complex represents an extreme case of specialisation, including at least six species that feed on Pocilloporidae, probably the original coralliophiline heritage. Similarly, the ancestor of the clade comprising *C. robillardi* and the five species ascribed to *C. fimbriata*, has been associated with the shallow-water Agariciidae since approximately 12–33 mya. Conversely, the shallow-water *Leptoconchus* spp. are estimated to have first evolved in association with Faviidae (13–23 mya), then underwent a broad host diversification in which one major lineage transitioned to Fungiidae (6–10 mya), while other lineages switched to Merulinidae or maintained their association with Faviidae (5–16 mya). The clade subtended by node 188, after an initial association with shallow-water Poritidae (around 18–20 mya), underwent a broad diversification in host associations, with extant species feeding on five different families.

The results obtained by the BAMM analysis of coralliophiline diversification rates across time did not highlight any core shift in diversification rates across the phylogeny of the subfamily (Fig. 5a). The shape of the rate-through-time plot suggests that the diversification rate of the family started decreasing at the beginning of the coralliophiline evolutionary history and continued to steadily slow down until the present day (Fig. 5b). The same pattern has been observed in another corallivorous gastropod lineage, the family Ovulidae (Nocella et al. 2024). As in the Ovulidae, we suggest that only during the initial stages of the evolution of the Coralliophilinae, a high diversification rate (∼0.15) existed; subsequently, this rate gradually declined in all lineages as time progressed. Following an initial burst of diversification, the progressive occupation of all available niches may result in a deceleration of the speciation rate within a density-dependent model (Moen and Morlon 2014). In our case, the initial high diversification rate could be linked to the emergence, in the ancestral coralliophilines, of the capability to exploit cnidarians as a trophic resource. The enduring association of Coralliophilinae with cnidarians has forged such robust relationships that have effectively constrained diversification rates for over 40 million years. In this context, in the only study to date available on macroevolutionary patterns of corallivorous gastropods (Nocella et al. 2024), the lack of distinct shifts in diversification rates within specific clades despite variations in species richness was instead associated with a pattern of slow, steady increase of diversification across the entire subfamily, linked to environmental variables. In Coralliophilinae this association started in shallow water with scleractinian hexacorals, with repeated instances of colonisations of deep-water habitats in several lineages. The origin of Scleractinia can be located from the Carboniferous to Silurian (324–447 mya, McFadden et al. 2021) followed by a rapid expansion and diversification across shallow marine environments during the Ladinian, about 240 mya (Frankowiak et al. 2023), thus providing a broad range of trophic niches available to corallivorous species. An ancestral exploitation of shallow-water hexacorals, with subsequent colonisations of deeper habitats, follows an onshore-offshore trend widely documented in marine fauna (Jacobs et al. 1998). However, we did not detect co-occurring shifts in depth and host.

Our results do not substantiate the existence of global co-evolutionary processes between coralliophiline gastropods and their cnidarian hosts. Instead, we found multiple unrelated switches from one cnidarian family to another. The exploited families of Scleractinia, Antipatharia, Zoantharia, Actiniaria and Malacalcyonacea, originated significantly earlier than Coralliophilinae (Lindner et al. 2008; McFadden et al. 2021). Therefore, neither the origin nor the diversification of the Coralliophilinae seems to have been strictly coupled with cnidarian evolution, suggesting the absence of a pervasive host–parasite co-evolutionary pattern. Similarly to what has been observed in the family Ovulidae (Nocella et al. 2024), the evolutionary trajectories of Coralliophilinae are better defined as a pattern of sequential evolution, where the evolution of the host may have influenced the evolution of the parasite, but not vice versa, as proposed in some association of phytophagous insects (Hardy and Otto 2014). The exploitation of the new niche offered by the corals represented a remarkable ecological opportunity, resulting in an ecological release that might have driven the evolution and diversification of corallivorous snails (Yoder et al. 2010), yet without a major bearing on cnidarian evolution. Nevertheless, we cannot exclude patterns of co-evolutionary processes within specific lineages, but their detection would require a denser sampling and a finer taxonomic identification of the cnidarian hosts at the genus or species level.

## Supplementary Information

Below is the link to the electronic supplementary material.

Supplementary file 1 (PDF 510 KB)

Supplementary file 2 (PDF 3293 KB)

Supplementary file 3 (PDF 78 KB)

Supplementary file 4 (XLSX 63 KB)

Supplementary file 5 (XLSX 10 KB)

Supplementary file 6 (XLSX 14 KB)
