# Supplementary material for: From coral reefs into the abyss: the evolution of corallivory in the Coralliophilinae (Neogastropoda, Muricidae)
Source: Coral Reefs. 2024 Aug 12;43(5):1285–302. doi: 10.1007/s00338-024-02537-1 (PMC11413129; doi:10.1007/s00338-024-02537-1)
Supplement: Supplementary file 1 — (PDF 510 KB) [file 338_2024_2537_MOESM1_ESM.pdf]

From coral reefs into the abyss: the evolution of corallivory in the Coralliophilinae (Neogastropoda, Muricidae)

*E. Nocella, G. Fassio, D. Zuccon, N. Puillandre, M.V. Modica, M. Oliverio

*Corresponding author: elisa.nocella@uniroma1.it
Department of Biology and Biotechnologies "Charles Darwin", Sapienza University of Rome, Rome, Italy
Department of Biology and Evolution of Marine Organisms, Stazione Zoologica Anton Dohrn, Napoli, Italy

Supporting Information

Figure S1: ASAP analysis of the COI dataset. Upper left, ASAP ten best partitions; upper central, ASAP ranked genetic distances; upper right, ASAP histogram of genetic distances; down left, voucher numbers; down right, the ten best partition identified by ASAP (each box correspond to one hypothetical species, the number of specimens included in each group is reported at the top of the box). On the top for each partition is reported the total number of hypothetical species found "Nb groups" and the ASAP score "Score". "W" = Relative Barcode Gap Width.

ASAP Results

The lower the score, the better the partition

Nb of subsets	asap-score		P-val (rank)	W (rank)	Treshold dist.	Text
67	5.50	●	1.32e-03 (2)	4.89e-05 (9)	0.120692	list csv
71	6.00	○	2.55e-01 (7)	5.33e-05 (5)	0.116113	list csv
* 118	12.50	○	5.21e-01 (11)	4.76e-05 (14)	0.039740	list csv
* 111	13.00	○	6.01e-01 (16)	4.87e-05 (10)	0.044442	list csv
* 117	13.00	○	7.57e-01 (22)	5.60e-05 (4)	0.040439	list csv
* 115	17.50	○	9.02e-01 (32)	5.98e-05 (3)	0.041254	list csv
75	19.50	○	6.69e-01 (19)	4.71e-05 (20)	0.112212	list csv
104	20.00	○	2.08e-01 (5)	3.31e-05 (35)	0.055829	list csv
79	21.00	○	6.31e-01 (17)	3.79e-05 (25)	0.108059	list csv
* 109	22.50	○	7.50e-01 (21)	3.81e-05 (24)	0.047667	list csv

Number of subsets in this table equals to the number of primary species hypotheses in the respective partition

Histogram of distances **[save]**

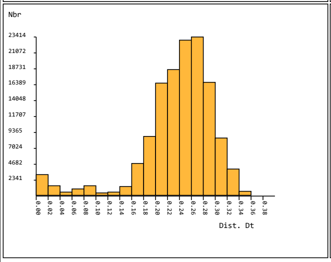

Ranked distances **[save]**

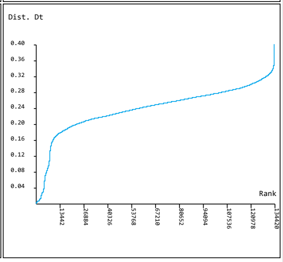

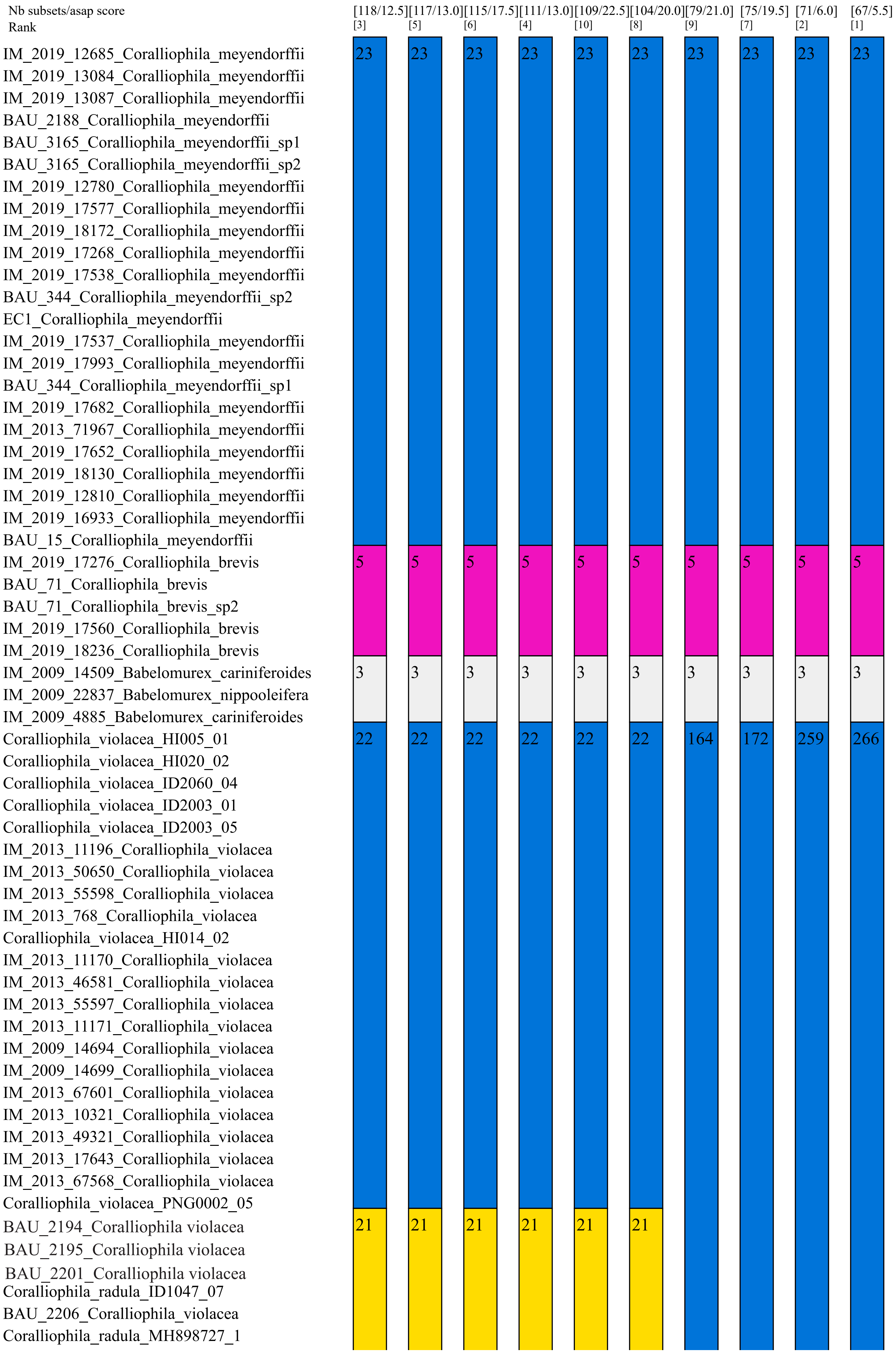
Nb subsets/asap score
Rank
[118/12.5] [3]
[117/13.0] [5]
[115/17.5] [6]
[111/13.0] [4]
[109/22.5] [10]
[104/20.0] [8]
[79/21.0] [9]
[75/19.5] [7]
[71/6.0] [2]
[67/5.5] [1]
IM_2019_12685_Coralliophila_meyendorffii
IM_2019_13084_Coralliophila_meyendorffii
IM_2019_13087_Coralliophila_meyendorffii
BAU_2188_Coralliophila_meyendorffii
BAU_3165_Coralliophila_meyendorffii_sp1
BAU_3165_Coralliophila_meyendorffii_sp2
IM_2019_12780_Coralliophila_meyendorffii
IM_2019_17577_Coralliophila_meyendorffii
IM_2019_18172_Coralliophila_meyendorffii
IM_2019_17268_Coralliophila_meyendorffii
IM_2019_17538_Coralliophila_meyendorffii
BAU_344_Coralliophila_meyendorffii_sp2
EC1_Coralliophila_meyendorffii
IM_2019_17537_Coralliophila_meyendorffii
IM_2019_17993_Coralliophila_meyendorffii
BAU_344_Coralliophila_meyendorffii_sp1
IM_2019_17682_Coralliophila_meyendorffii
IM_2013_71967_Coralliophila_meyendorffii
IM_2019_17652_Coralliophila_meyendorffii
IM_2019_18130_Coralliophila_meyendorffii
IM_2019_12810_Coralliophila_meyendorffii
IM_2019_16933_Coralliophila_meyendorffii
BAU_15_Coralliophila_meyendorffii
IM_2019_17276_Coralliophila_brevis
BAU_71_Coralliophila_brevis
BAU_71_Coralliophila_brevis_sp2
IM_2019_17560_Coralliophila_brevis
IM_2019_18236_Coralliophila_brevis
IM_2009_14509_Babelomurex_cariniferoides
IM_2009_22837_Babelomurex_nippooleifera
IM_2009_4885_Babelomurex_cariniferoides
Coralliophila_violacea_HI005_01
Coralliophila_violacea_HI020_02
Coralliophila_violacea_ID2060_04
Coralliophila_violacea_ID2003_01
Coralliophila_violacea_ID2003_05
IM_2013_11196_Coralliophila_violacea
IM_2013_50650_Coralliophila_violacea
IM_2013_55598_Coralliophila_violacea
IM_2013_768_Coralliophila_violacea
Coralliophila_violacea_HI014_02
IM_2013_11170_Coralliophila_violacea
IM_2013_46581_Coralliophila_violacea
IM_2013_55597_Coralliophila_violacea
IM_2013_11171_Coralliophila_violacea
IM_2009_14694_Coralliophila_violacea
IM_2009_14699_Coralliophila_violacea
IM_2013_67601_Coralliophila_violacea
IM_2013_10321_Coralliophila_violacea
IM_2013_49321_Coralliophila_violacea
IM_2013_17643_Coralliophila_violacea
IM_2013_67568_Coralliophila_violacea
Coralliophila_violacea_PNG0002_05
BAU_2194_Coralliophila violacea
BAU_2195_Coralliophila violacea
BAU_2201_Coralliophila violacea
Coralliophila_radula_ID1047_07
BAU_2206_Coralliophila_violacea
Coralliophila_radula_MH898727_1
23 23 23 23 23 23 23 23 23 23
5 5 5 5 5 5 5 5 5 5
3 3 3 3 3 3 3 3 3 3
22 22 22 22 22 22 164 172 259 266
21 21 21 21 21 21

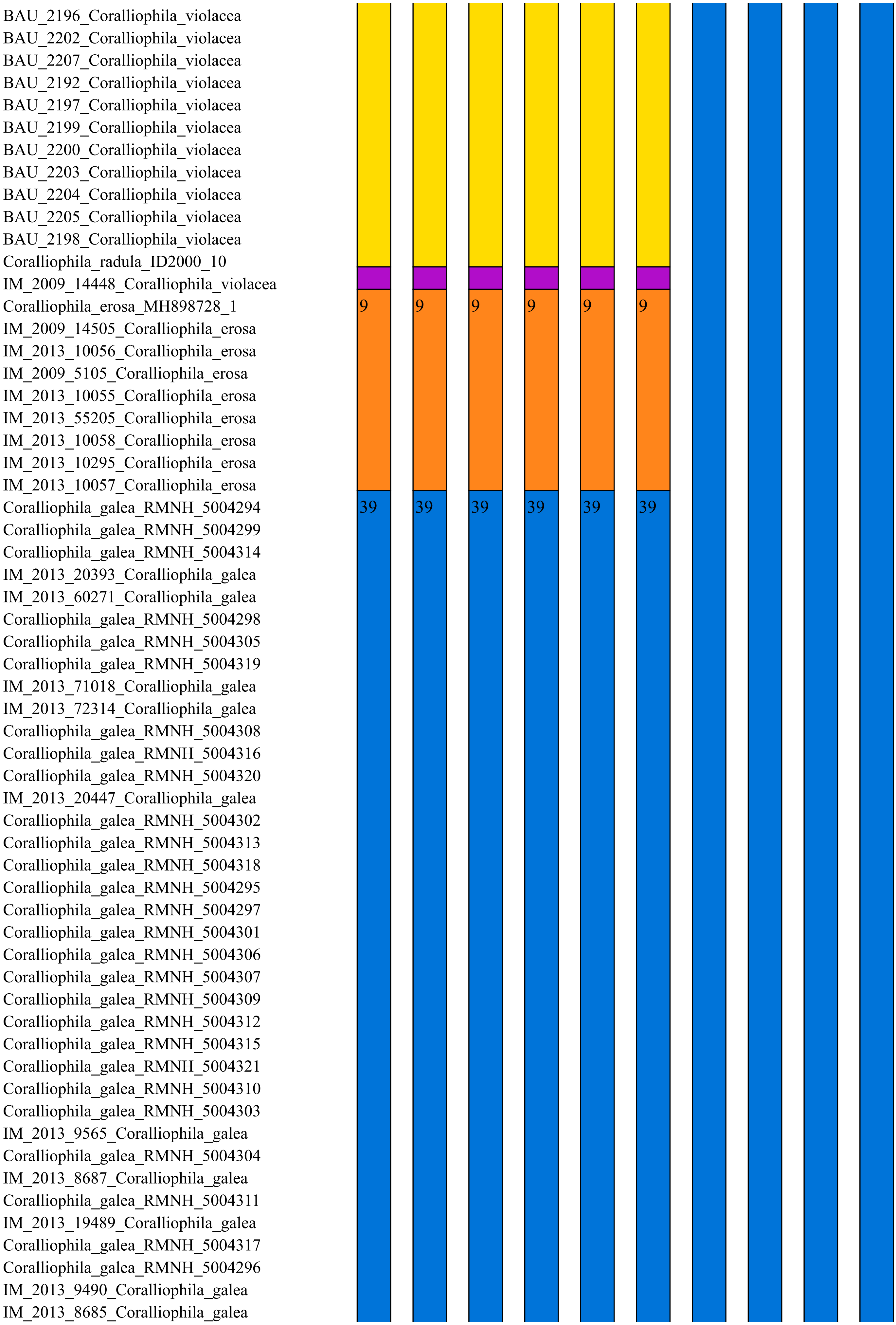
BAU_2196_Coralliophila_violacea
BAU_2202_Coralliophila_violacea
BAU_2207_Coralliophila_violacea
BAU_2192_Coralliophila_violacea
BAU_2197_Coralliophila_violacea
BAU_2199_Coralliophila_violacea
BAU_2200_Coralliophila_violacea
BAU_2203_Coralliophila_violacea
BAU_2204_Coralliophila_violacea
BAU_2205_Coralliophila_violacea
BAU_2198_Coralliophila_violacea
Coralliophila_radula_ID2000_10
IM_2009_14448_Coralliophila_violacea
Coralliophila_erosa_MH898728_1
IM_2009_14505_Coralliophila_erosa
IM_2013_10056_Coralliophila_erosa
IM_2009_5105_Coralliophila_erosa
IM_2013_10055_Coralliophila_erosa
IM_2013_55205_Coralliophila_erosa
IM_2013_10058_Coralliophila_erosa
IM_2013_10295_Coralliophila_erosa
IM_2013_10057_Coralliophila_erosa
Coralliophila_galea_RMNH_5004294
Coralliophila_galea_RMNH_5004299
Coralliophila_galea_RMNH_5004314
IM_2013_20393_Coralliophila_galea
IM_2013_60271_Coralliophila_galea
Coralliophila_galea_RMNH_5004298
Coralliophila_galea_RMNH_5004305
Coralliophila_galea_RMNH_5004319
IM_2013_71018_Coralliophila_galea
IM_2013_72314_Coralliophila_galea
Coralliophila_galea_RMNH_5004308
Coralliophila_galea_RMNH_5004316
Coralliophila_galea_RMNH_5004320
IM_2013_20447_Coralliophila_galea
Coralliophila_galea_RMNH_5004302
Coralliophila_galea_RMNH_5004313
Coralliophila_galea_RMNH_5004318
Coralliophila_galea_RMNH_5004295
Coralliophila_galea_RMNH_5004297
Coralliophila_galea_RMNH_5004301
Coralliophila_galea_RMNH_5004306
Coralliophila_galea_RMNH_5004307
Coralliophila_galea_RMNH_5004309
Coralliophila_galea_RMNH_5004312
Coralliophila_galea_RMNH_5004315
Coralliophila_galea_RMNH_5004321
Coralliophila_galea_RMNH_5004310
Coralliophila_galea_RMNH_5004303
IM_2013_9565_Coralliophila_galea
Coralliophila_galea_RMNH_5004304
IM_2013_8687_Coralliophila_galea
Coralliophila_galea_RMNH_5004311
IM_2013_19489_Coralliophila_galea
Coralliophila_galea_RMNH_5004317
Coralliophila_galea_RMNH_5004296
IM_2013_9490_Coralliophila_galea
IM_2013_8685_Coralliophila_galea
9
9
9
9
9
9
39
39
39
39
39
39

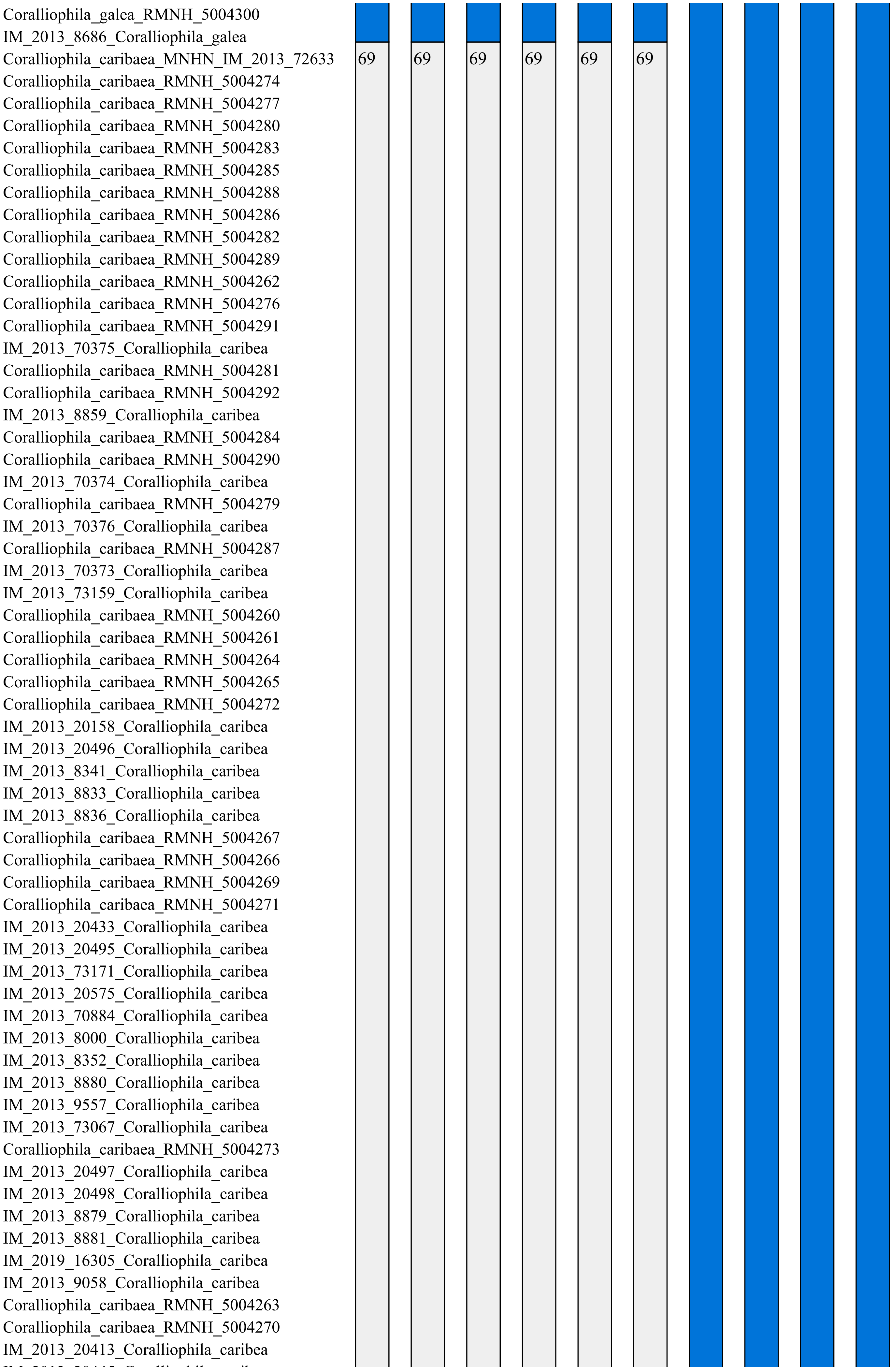
Coralliophila_galea_RMNH_5004300
IM_2013_8686_Coralliophila_galea
Coralliophila_caribaea_MNHN_IM_2013_72633
69
69
69
69
69
69
Coralliophila_caribaea_RMNH_5004274
Coralliophila_caribaea_RMNH_5004277
Coralliophila_caribaea_RMNH_5004280
Coralliophila_caribaea_RMNH_5004283
Coralliophila_caribaea_RMNH_5004285
Coralliophila_caribaea_RMNH_5004288
Coralliophila_caribaea_RMNH_5004286
Coralliophila_caribaea_RMNH_5004282
Coralliophila_caribaea_RMNH_5004289
Coralliophila_caribaea_RMNH_5004262
Coralliophila_caribaea_RMNH_5004276
Coralliophila_caribaea_RMNH_5004291
IM_2013_70375_Coralliophila_caribea
Coralliophila_caribaea_RMNH_5004281
Coralliophila_caribaea_RMNH_5004292
IM_2013_8859_Coralliophila_caribea
Coralliophila_caribaea_RMNH_5004284
Coralliophila_caribaea_RMNH_5004290
IM_2013_70374_Coralliophila_caribea
Coralliophila_caribaea_RMNH_5004279
IM_2013_70376_Coralliophila_caribea
Coralliophila_caribaea_RMNH_5004287
IM_2013_70373_Coralliophila_caribea
IM_2013_73159_Coralliophila_caribea
Coralliophila_caribaea_RMNH_5004260
Coralliophila_caribaea_RMNH_5004261
Coralliophila_caribaea_RMNH_5004264
Coralliophila_caribaea_RMNH_5004265
Coralliophila_caribaea_RMNH_5004272
IM_2013_20158_Coralliophila_caribea
IM_2013_20496_Coralliophila_caribea
IM_2013_8341_Coralliophila_caribea
IM_2013_8833_Coralliophila_caribea
IM_2013_8836_Coralliophila_caribea
Coralliophila_caribaea_RMNH_5004267
Coralliophila_caribaea_RMNH_5004266
Coralliophila_caribaea_RMNH_5004269
Coralliophila_caribaea_RMNH_5004271
IM_2013_20433_Coralliophila_caribea
IM_2013_20495_Coralliophila_caribea
IM_2013_73171_Coralliophila_caribea
IM_2013_20575_Coralliophila_caribea
IM_2013_70884_Coralliophila_caribea
IM_2013_8000_Coralliophila_caribea
IM_2013_8352_Coralliophila_caribea
IM_2013_8880_Coralliophila_caribea
IM_2013_9557_Coralliophila_caribea
IM_2013_73067_Coralliophila_caribea
Coralliophila_caribaea_RMNH_5004273
IM_2013_20497_Coralliophila_caribea
IM_2013_20498_Coralliophila_caribea
IM_2013_8879_Coralliophila_caribea
IM_2013_8881_Coralliophila_caribea
IM_2019_16305_Coralliophila_caribea
IM_2013_9058_Coralliophila_caribea
Coralliophila_caribaea_RMNH_5004263
Coralliophila_caribaea_RMNH_5004270
IM_2013_20413_Coralliophila_caribea

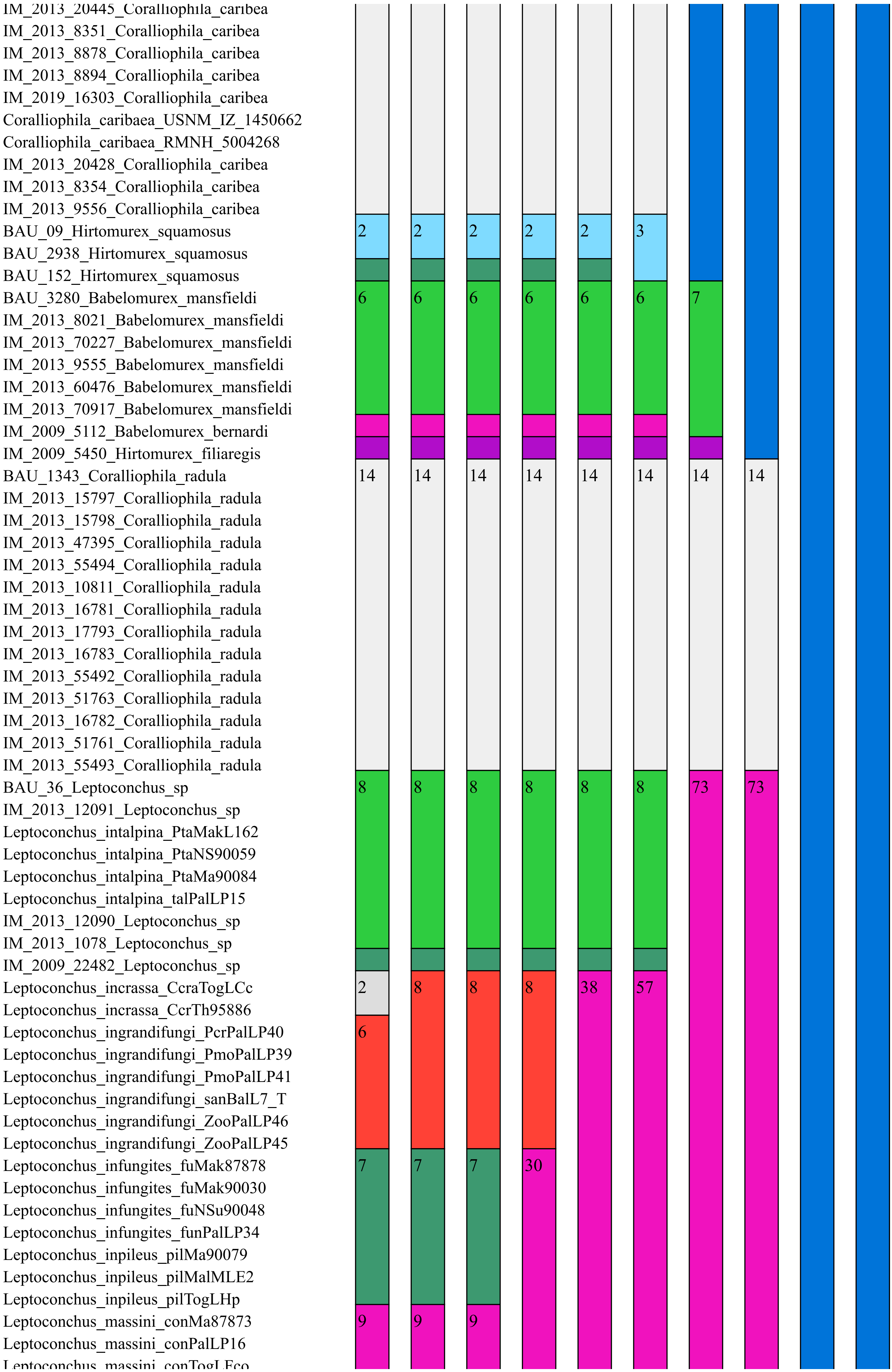
IM_2013_20445_Coralliophila_caribea
IM_2013_8351_Coralliophila_caribea
IM_2013_8878_Coralliophila_caribea
IM_2013_8894_Coralliophila_caribea
IM_2019_16303_Coralliophila_caribea
Coralliophila_caribaea_USNM_IZ_1450662
Coralliophila_caribaea_RMNH_5004268
IM_2013_20428_Coralliophila_caribea
IM_2013_8354_Coralliophila_caribea
IM_2013_9556_Coralliophila_caribea
BAU_09_Hirtomurex_squamosus
BAU_2938_Hirtomurex_squamosus
BAU_152_Hirtomurex_squamosus
BAU_3280_Babelomurex_mansfieldi
IM_2013_8021_Babelomurex_mansfieldi
IM_2013_70227_Babelomurex_mansfieldi
IM_2013_9555_Babelomurex_mansfieldi
IM_2013_60476_Babelomurex_mansfieldi
IM_2013_70917_Babelomurex_mansfieldi
IM_2009_5112_Babelomurex_bernardi
IM_2009_5450_Hirtomurex_filiaregis
BAU_1343_Coralliophila_radula
IM_2013_15797_Coralliophila_radula
IM_2013_15798_Coralliophila_radula
IM_2013_47395_Coralliophila_radula
IM_2013_55494_Coralliophila_radula
IM_2013_10811_Coralliophila_radula
IM_2013_16781_Coralliophila_radula
IM_2013_17793_Coralliophila_radula
IM_2013_16783_Coralliophila_radula
IM_2013_55492_Coralliophila_radula
IM_2013_51763_Coralliophila_radula
IM_2013_16782_Coralliophila_radula
IM_2013_51761_Coralliophila_radula
IM_2013_55493_Coralliophila_radula
BAU_36_Leptoconchus_sp
IM_2013_12091_Leptoconchus_sp
Leptoconchus_intalpina_PtaMakL162
Leptoconchus_intalpina_PtaNS90059
Leptoconchus_intalpina_PtaMa90084
Leptoconchus_intalpina_talPalLP15
IM_2013_12090_Leptoconchus_sp
IM_2013_1078_Leptoconchus_sp
IM_2009_22482_Leptoconchus_sp
Leptoconchus_incrassa_CcraTogLCc
Leptoconchus_incrassa_CcrTh95886
Leptoconchus_ingrandifungi_PcrPalLP40
Leptoconchus_ingrandifungi_PmoPalLP39
Leptoconchus_ingrandifungi_PmoPalLP41
Leptoconchus_ingrandifungi_sanBalL7_T
Leptoconchus_ingrandifungi_ZooPalLP46
Leptoconchus_ingrandifungi_ZooPalLP45
Leptoconchus_infungites_fuMak87878
Leptoconchus_infungites_fuMak90030
Leptoconchus_infungites_fuNSu90048
Leptoconchus_infungites_funPalLP34
Leptoconchus_inpileus_pilMa90079
Leptoconchus_inpileus_pilMalMLE2
Leptoconchus_inpileus_pilTogLHp
Leptoconchus_massini_conMa87873
Leptoconchus_massini_conPalLP16
2 2 2 2 2 3
6 6 6 6 6 6 7
14 14 14 14 14 14 14 14
8 8 8 8 8 8 73 73
2 8 8 8 38 57
6
7 7 7 30
9 9 9

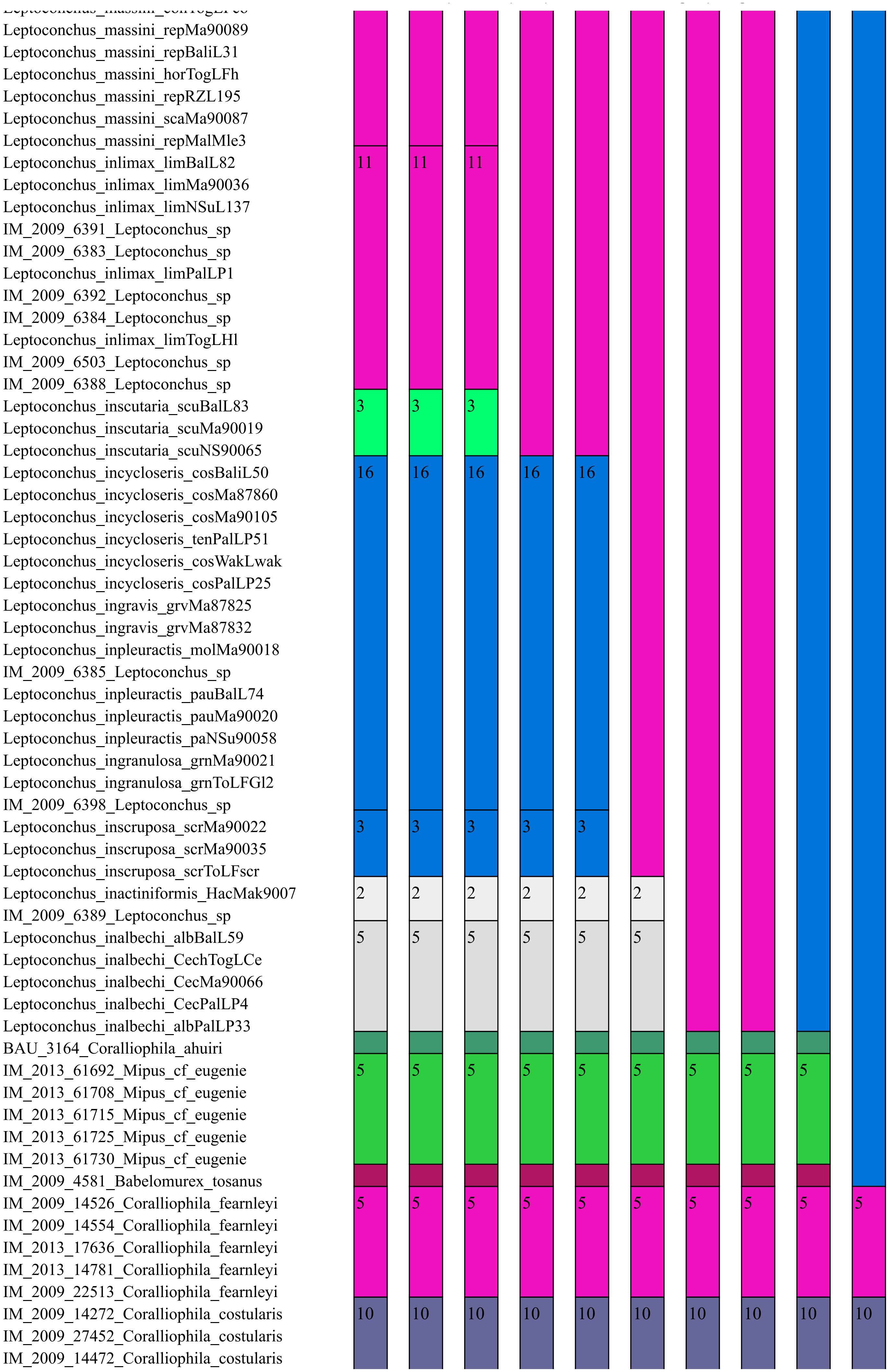
Leptoconchus_massini_repMa90089
Leptoconchus_massini_repBaliL31
Leptoconchus_massini_horTogLFh
Leptoconchus_massini_repRZL195
Leptoconchus_massini_scaMa90087
Leptoconchus_massini_repMalMle3
Leptoconchus_inlimax_limBalL82
Leptoconchus_inlimax_limMa90036
Leptoconchus_inlimax_limNSuL137
IM_2009_6391_Leptoconchus_sp
IM_2009_6383_Leptoconchus_sp
Leptoconchus_inlimax_limPalLP1
IM_2009_6392_Leptoconchus_sp
IM_2009_6384_Leptoconchus_sp
Leptoconchus_inlimax_limTogLHl
IM_2009_6503_Leptoconchus_sp
IM_2009_6388_Leptoconchus_sp
Leptoconchus_inscutaria_scuBalL83
Leptoconchus_inscutaria_scuMa90019
Leptoconchus_inscutaria_scuNS90065
Leptoconchus_incycloseris_cosBaliL50
Leptoconchus_incycloseris_cosMa87860
Leptoconchus_incycloseris_cosMa90105
Leptoconchus_incycloseris_tenPalLP51
Leptoconchus_incycloseris_cosWakLwak
Leptoconchus_incycloseris_cosPalLP25
Leptoconchus_ingravis_grvMa87825
Leptoconchus_ingravis_grvMa87832
Leptoconchus_inpleuractis_molMa90018
IM_2009_6385_Leptoconchus_sp
Leptoconchus_inpleuractis_pauBalL74
Leptoconchus_inpleuractis_pauMa90020
Leptoconchus_inpleuractis_paNSu90058
Leptoconchus_ingranulosa_grnMa90021
Leptoconchus_ingranulosa_grnToLFGl2
IM_2009_6398_Leptoconchus_sp
Leptoconchus_inscruposa_scrMa90022
Leptoconchus_inscruposa_scrMa90035
Leptoconchus_inscruposa_scrToLFscr
Leptoconchus_inactiniformis_HacMak9007
IM_2009_6389_Leptoconchus_sp
Leptoconchus_inalbechi_albBalL59
Leptoconchus_inalbechi_CechTogLCe
Leptoconchus_inalbechi_CecMa90066
Leptoconchus_inalbechi_CecPalLP4
Leptoconchus_inalbechi_albPalLP33
BAU_3164_Coralliophila_ahuiri
IM_2013_61692_Mipus_cf_eugenie
IM_2013_61708_Mipus_cf_eugenie
IM_2013_61715_Mipus_cf_eugenie
IM_2013_61725_Mipus_cf_eugenie
IM_2013_61730_Mipus_cf_eugenie
IM_2009_4581_Babelomurex_tosanus
IM_2009_14526_Coralliophila_fearnleyi
IM_2009_14554_Coralliophila_fearnleyi
IM_2013_17636_Coralliophila_fearnleyi
IM_2013_14781_Coralliophila_fearnleyi
IM_2009_22513_Coralliophila_fearnleyi
IM_2009_14272_Coralliophila_costularis
IM_2009_27452_Coralliophila_costularis
IM_2009_14472_Coralliophila_costularis
11
3
16
3
2
5
5
5
10

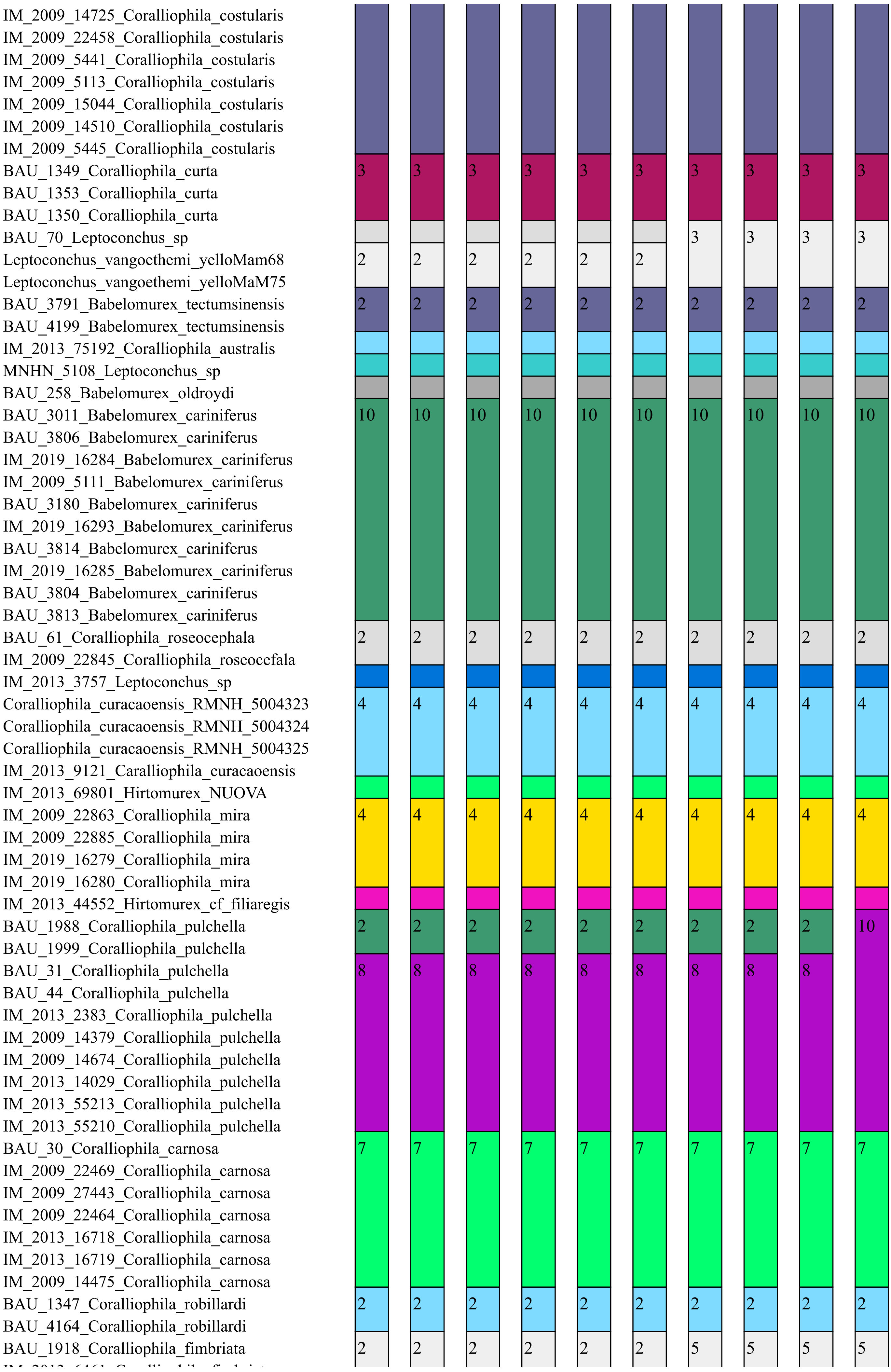

IM_2009_14725_Coralliophila_costularis
IM_2009_22458_Coralliophila_costularis
IM_2009_5441_Coralliophila_costularis
IM_2009_5113_Coralliophila_costularis
IM_2009_15044_Coralliophila_costularis
IM_2009_14510_Coralliophila_costularis
IM_2009_5445_Coralliophila_costularis
BAU_1349_Coralliophila_curta
BAU_1353_Coralliophila_curta
BAU_1350_Coralliophila_curta
BAU_70_Leptoconchus_sp
Leptoconchus_vangoethemi_yelloMam68
Leptoconchus_vangoethemi_yelloMaM75
BAU_3791_Babelomurex_tectumsinensis
BAU_4199_Babelomurex_tectumsinensis
IM_2013_75192_Coralliophila_australis
MNHN_5108_Leptoconchus_sp
BAU_258_Babelomurex_oldroydi
BAU_3011_Babelomurex_cariniferus
BAU_3806_Babelomurex_cariniferus
IM_2019_16284_Babelomurex_cariniferus
IM_2009_5111_Babelomurex_cariniferus
BAU_3180_Babelomurex_cariniferus
IM_2019_16293_Babelomurex_cariniferus
BAU_3814_Babelomurex_cariniferus
IM_2019_16285_Babelomurex_cariniferus
BAU_3804_Babelomurex_cariniferus
BAU_3813_Babelomurex_cariniferus
BAU_61_Coralliophila_roseocephala
IM_2009_22845_Coralliophila_roseocefala
IM_2013_3757_Leptoconchus_sp
Coralliophila_curacaoensis_RMNH_5004323
Coralliophila_curacaoensis_RMNH_5004324
Coralliophila_curacaoensis_RMNH_5004325
IM_2013_9121_Carallіophila_curacaoensis
IM_2013_69801_Hirtomurex_NUOVA
IM_2009_22863_Coralliophila_mira
IM_2009_22885_Coralliophila_mira
IM_2019_16279_Coralliophila_mira
IM_2019_16280_Coralliophila_mira
IM_2013_44552_Hirtomurex_cf_filiaregis
BAU_1988_Coralliophila_pulchella
BAU_1999_Coralliophila_pulchella
BAU_31_Coralliophila_pulchella
BAU_44_Coralliophila_pulchella
IM_2013_2383_Coralliophila_pulchella
IM_2009_14379_Coralliophila_pulchella
IM_2009_14674_Coralliophila_pulchella
IM_2013_14029_Coralliophila_pulchella
IM_2013_55213_Coralliophila_pulchella
IM_2013_55210_Coralliophila_pulchella
BAU_30_Coralliophila_carnosa
IM_2009_22469_Coralliophila_carnosa
IM_2009_27443_Coralliophila_carnosa
IM_2009_22464_Coralliophila_carnosa
IM_2013_16718_Coralliophila_carnosa
IM_2013_16719_Coralliophila_carnosa
IM_2009_14475_Coralliophila_carnosa
BAU_1347_Coralliophila_robillardi
BAU_4164_Coralliophila_robillardi
BAU_1918_Coralliophila_fimbriata

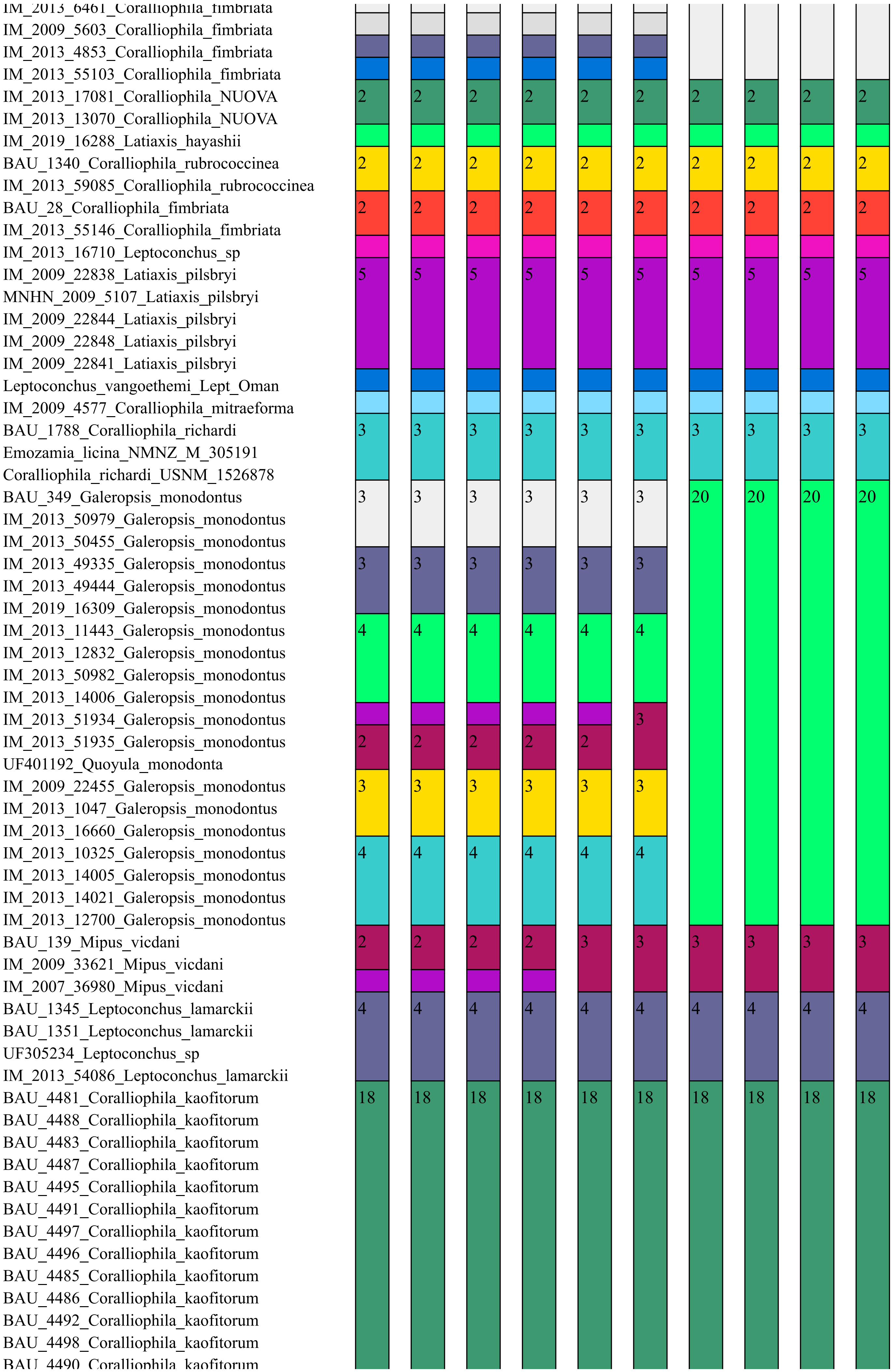
IM_2013_6461_Coralliophila_fimbriata
IM_2009_5603_Coralliophila_fimbriata
IM_2013_4853_Coralliophila_fimbriata
IM_2013_55103_Coralliophila_fimbriata
IM_2013_17081_Coralliophila_NUOVA
IM_2013_13070_Coralliophila_NUOVA
IM_2019_16288_Latiaxis_hayashii
BAU_1340_Coralliophila_rubrococcinea
IM_2013_59085_Coralliophila_rubrococcinea
BAU_28_Coralliophila_fimbriata
IM_2013_55146_Coralliophila_fimbriata
IM_2013_16710_Leptoconchus_sp
IM_2009_22838_Latiaxis_pilsbryi
MNHN_2009_5107_Latiaxis_pilsbryi
IM_2009_22844_Latiaxis_pilsbryi
IM_2009_22848_Latiaxis_pilsbryi
IM_2009_22841_Latiaxis_pilsbryi
Leptoconchus_vangoethemi_Lept_Oman
IM_2009_4577_Coralliophila_mitraeforma
BAU_1788_Coralliophila_richardi
Emozamia_licina_NMNZ_M_305191
Coralliophila_richardi_USNM_1526878
BAU_349_Galeropsis_monodontus
IM_2013_50979_Galeropsis_monodontus
IM_2013_50455_Galeropsis_monodontus
IM_2013_49335_Galeropsis_monodontus
IM_2013_49444_Galeropsis_monodontus
IM_2019_16309_Galeropsis_monodontus
IM_2013_11443_Galeropsis_monodontus
IM_2013_12832_Galeropsis_monodontus
IM_2013_50982_Galeropsis_monodontus
IM_2013_14006_Galeropsis_monodontus
IM_2013_51934_Galeropsis_monodontus
IM_2013_51935_Galeropsis_monodontus
UF401192_Quoyula_monodonta
IM_2009_22455_Galeropsis_monodontus
IM_2013_1047_Galeropsis_monodontus
IM_2013_16660_Galeropsis_monodontus
IM_2013_10325_Galeropsis_monodontus
IM_2013_14005_Galeropsis_monodontus
IM_2013_14021_Galeropsis_monodontus
IM_2013_12700_Galeropsis_monodontus
BAU_139_Mipus_vicdani
IM_2009_33621_Mipus_vicdani
IM_2007_36980_Mipus_vicdani
BAU_1345_Leptoconchus_lamarckii
BAU_1351_Leptoconchus_lamarckii
UF305234_Leptoconchus_sp
IM_2013_54086_Leptoconchus_lamarckii
BAU_4481_Coralliophila_kaofitorum
BAU_4488_Coralliophila_kaofitorum
BAU_4483_Coralliophila_kaofitorum
BAU_4487_Coralliophila_kaofitorum
BAU_4495_Coralliophila_kaofitorum
BAU_4491_Coralliophila_kaofitorum
BAU_4497_Coralliophila_kaofitorum
BAU_4496_Coralliophila_kaofitorum
BAU_4485_Coralliophila_kaofitorum
BAU_4486_Coralliophila_kaofitorum
BAU_4492_Coralliophila_kaofitorum
BAU_4498_Coralliophila_kaofitorum
BAU_4490_Coralliophila_kaofitorum

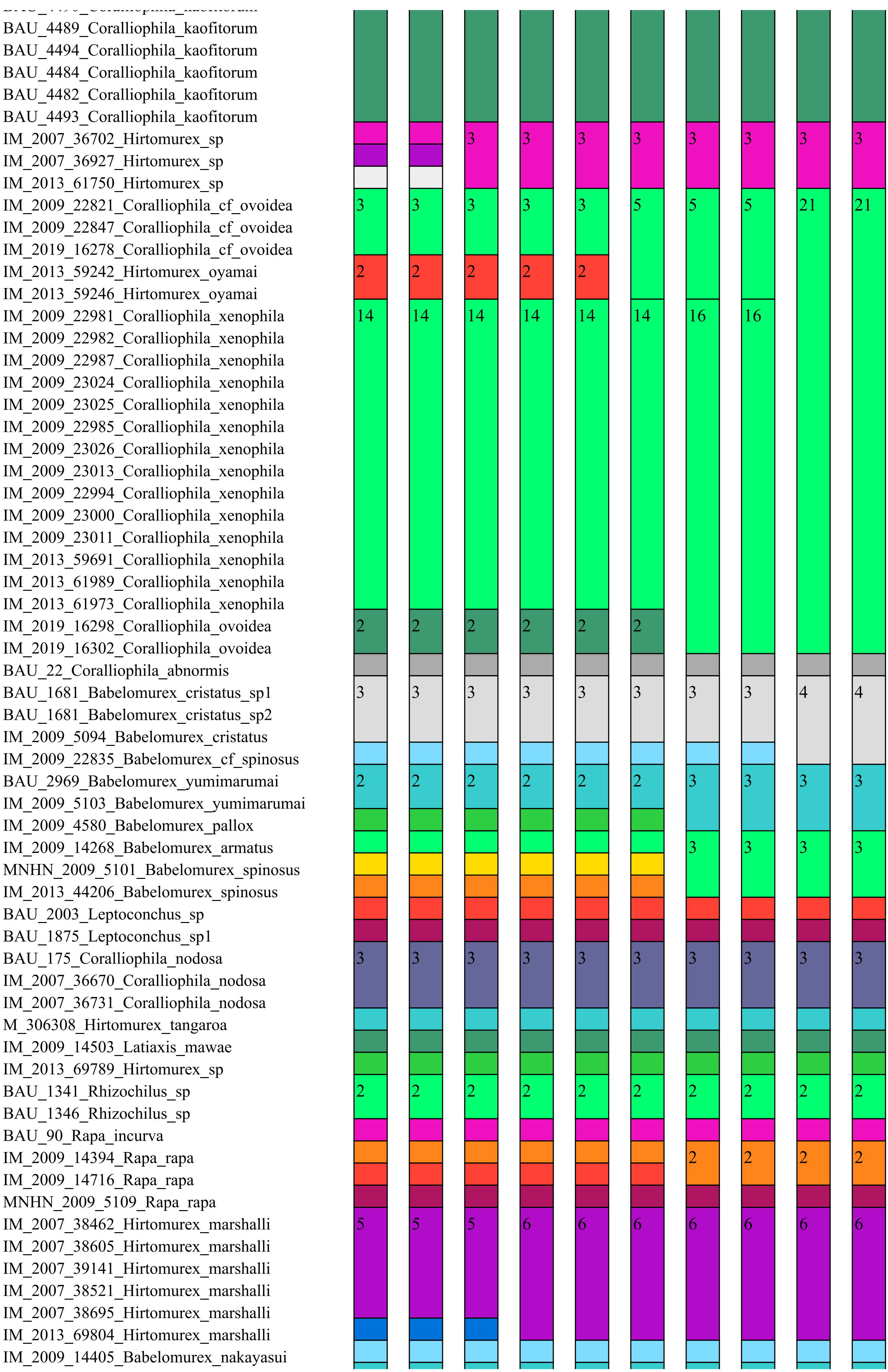
BAU_4489_Coralliophila_kaofitorum
BAU_4494_Coralliophila_kaofitorum
BAU_4484_Coralliophila_kaofitorum
BAU_4482_Coralliophila_kaofitorum
BAU_4493_Coralliophila_kaofitorum
IM_2007_36702_Hirtomurex_sp
IM_2007_36927_Hirtomurex_sp
IM_2013_61750_Hirtomurex_sp
IM_2009_22821_Coralliophila_cf_ovoidea
IM_2009_22847_Coralliophila_cf_ovoidea
IM_2019_16278_Coralliophila_cf_ovoidea
IM_2013_59242_Hirtomurex_oyamai
IM_2013_59246_Hirtomurex_oyamai
IM_2009_22981_Coralliophila_xenophila
IM_2009_22982_Coralliophila_xenophila
IM_2009_22987_Coralliophila_xenophila
IM_2009_23024_Coralliophila_xenophila
IM_2009_23025_Coralliophila_xenophila
IM_2009_22985_Coralliophila_xenophila
IM_2009_23026_Coralliophila_xenophila
IM_2009_23013_Coralliophila_xenophila
IM_2009_22994_Coralliophila_xenophila
IM_2009_23000_Coralliophila_xenophila
IM_2009_23011_Coralliophila_xenophila
IM_2013_59691_Coralliophila_xenophila
IM_2013_61989_Coralliophila_xenophila
IM_2013_61973_Coralliophila_xenophila
IM_2019_16298_Coralliophila_ovoidea
IM_2019_16302_Coralliophila_ovoidea
BAU_22_Coralliophila_abnormis
BAU_1681_Babelomurex_cristatus_sp1
BAU_1681_Babelomurex_cristatus_sp2
IM_2009_5094_Babelomurex_cristatus
IM_2009_22835_Babelomurex_cf_spinosus
BAU_2969_Babelomurex_yumimarumai
IM_2009_5103_Babelomurex_yumimarumai
IM_2009_4580_Babelomurex_pallox
IM_2009_14268_Babelomurex_armatus
MNHN_2009_5101_Babelomurex_spinosus
IM_2013_44206_Babelomurex_spinosus
BAU_2003_Leptoconchus_sp
BAU_1875_Leptoconchus_sp1
BAU_175_Coralliophila_nodosa
IM_2007_36670_Coralliophila_nodosa
IM_2007_36731_Coralliophila_nodosa
M_306308_Hirtomurex_tangaroa
IM_2009_14503_Latiaxis_mawae
IM_2013_69789_Hirtomurex_sp
BAU_1341_Rhizochilus_sp
BAU_1346_Rhizochilus_sp
BAU_90_Rapa_incurva
IM_2009_14394_Rapa_rapa
IM_2009_14716_Rapa_rapa
MNHN_2009_5109_Rapa_rapa
IM_2007_38462_Hirtomurex_marshalli
IM_2007_38605_Hirtomurex_marshalli
IM_2007_39141_Hirtomurex_marshalli
IM_2007_38521_Hirtomurex_marshalli
IM_2007_38695_Hirtomurex_marshalli
IM_2013_69804_Hirtomurex_marshalli
IM_2009_14405_Babelomurex_nakayasui

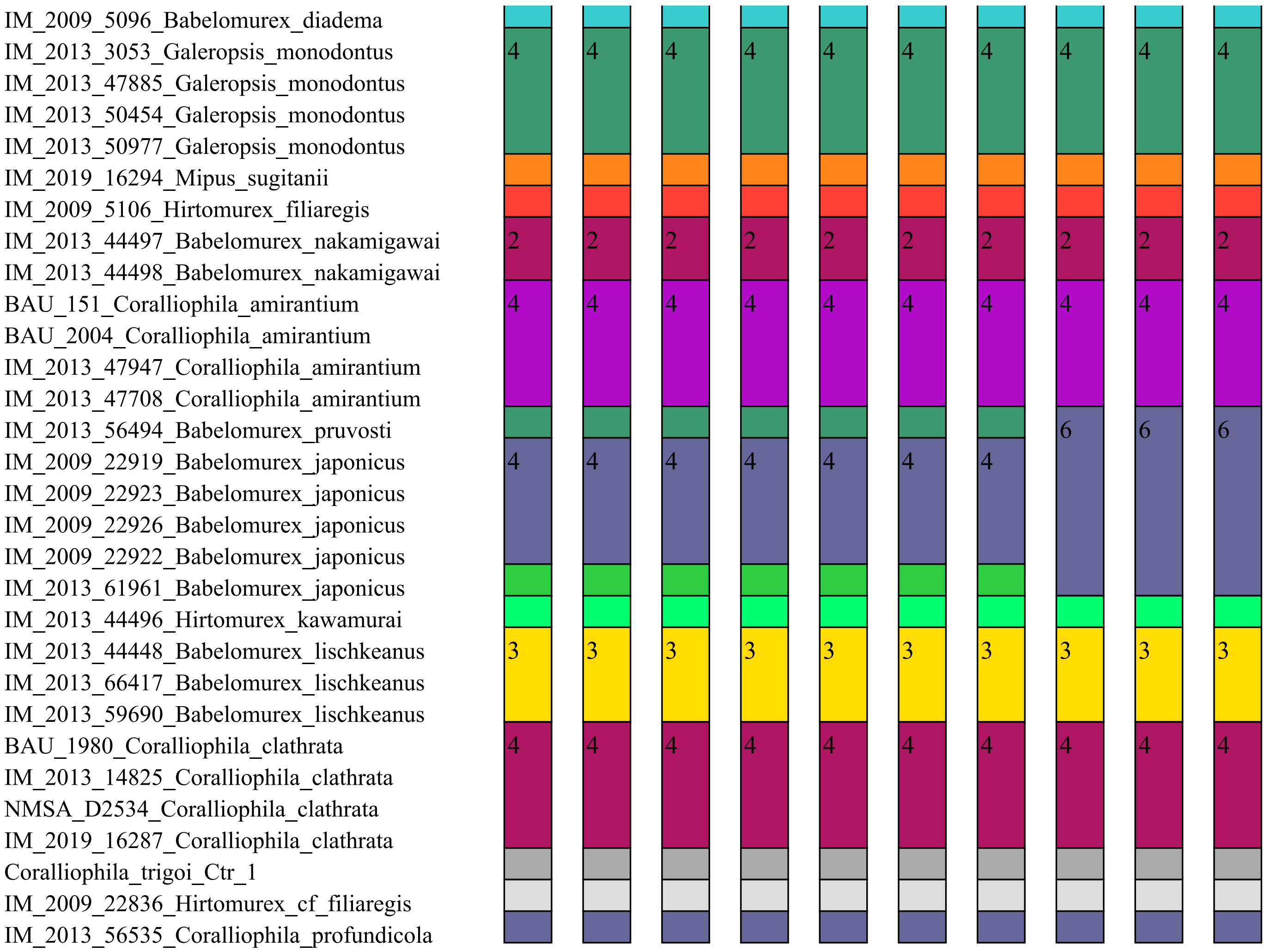
IM_2009_5096_Babelomurex_diadema
IM_2013_3053_Galeropsis_monodontus
IM_2013_47885_Galeropsis_monodontus
IM_2013_50454_Galeropsis_monodontus
IM_2013_50977_Galeropsis_monodontus
IM_2019_16294_Mipus_sugitanii
IM_2009_5106_Hirtomurex_filiaregis
IM_2013_44497_Babelomurex_nakamigawai
IM_2013_44498_Babelomurex_nakamigawai
BAU_151_Coralliophila_amirantium
BAU_2004_Coralliophila_amirantium
IM_2013_47947_Coralliophila_amirantium
IM_2013_47708_Coralliophila_amirantium
IM_2013_56494_Babelomurex_pruvosti
IM_2009_22919_Babelomurex_japonicus
IM_2009_22923_Babelomurex_japonicus
IM_2009_22926_Babelomurex_japonicus
IM_2009_22922_Babelomurex_japonicus
IM_2013_61961_Babelomurex_japonicus
IM_2013_44496_Hirtomurex_kawamurai
IM_2013_44448_Babelomurex_lischkeanus
IM_2013_66417_Babelomurex_lischkeanus
IM_2013_59690_Babelomurex_lischkeanus
BAU_1980_Coralliophila_clathrata
IM_2013_14825_Coralliophila_clathrata
NMSA_D2534_Coralliophila_clathrata
IM_2019_16287_Coralliophila_clathrata
Coralliophila_trigoi_Ctr_1
IM_2009_22836_Hirtomurex_cf_filiaregis
IM_2013_56535_Coralliophila_profundicola
