# Supplementary material for: From coral reefs into the abyss: the evolution of corallivory in the Coralliophilinae (Neogastropoda, Muricidae)
Source: Coral Reefs. 2024 Aug 12;43(5):1285–302. doi: 10.1007/s00338-024-02537-1 (PMC11413129; doi:10.1007/s00338-024-02537-1)

From coral reefs into the abyss: the evolution of corallivory in the Coralliophilinae (Neogastropoda, Muricidae)

[*] E. Nocella, G. Fassio, D. Zuccon, N. Puillandre, M.V. Modica, M. Oliverio

*Corresponding author: elisa.nocella@uniroma1.it
Department of Biology and Biotechnologies "Charles Darwin", Sapienza University of Rome, Rome, Italy
Department of Biology and Evolution of Marine Organisms, Stazione Zoologica Anton Dohrn, Napoli, Italy

Supplementary Information: Figures S2-S9

Figure S2. Maximum Likelihood phylogenetic inference on the COI-16S-ITS2 combined dataset. Numbers at nodes indicate ultrafast bootstrap values.

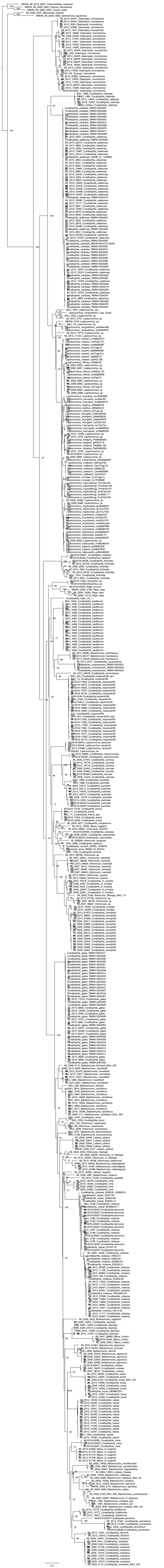

Figure S3. Maximum Likelihood phylogenetic inference on the COI dataset. Numbers at nodes indicate ultrafast bootstrap values.

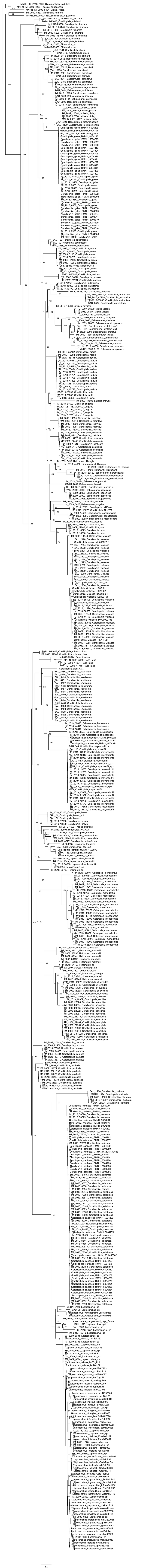

Figure S4. Maximum Likelihood phylogenetic inference on the 16S combined dataset. Numbers at nodes indicate ultrafast bootstrap values.

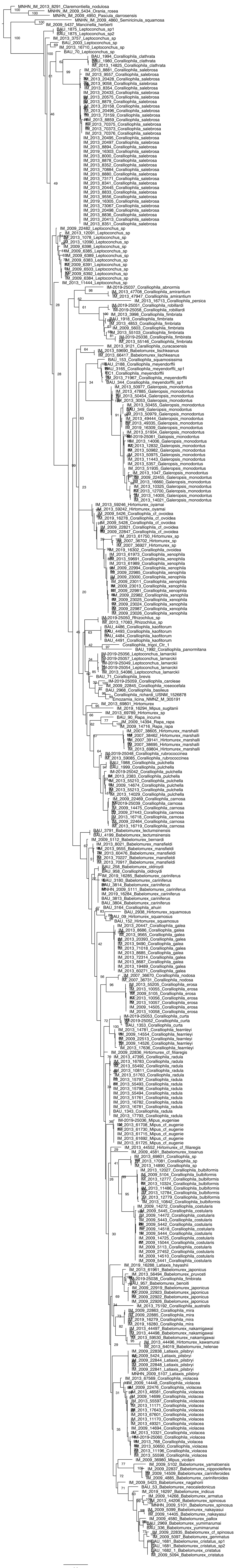

Figure S5. Maximum Likelihood phylogenetic inference on the ITS2 dataset. Numbers at nodes indicate ultrafast bootstrap values.

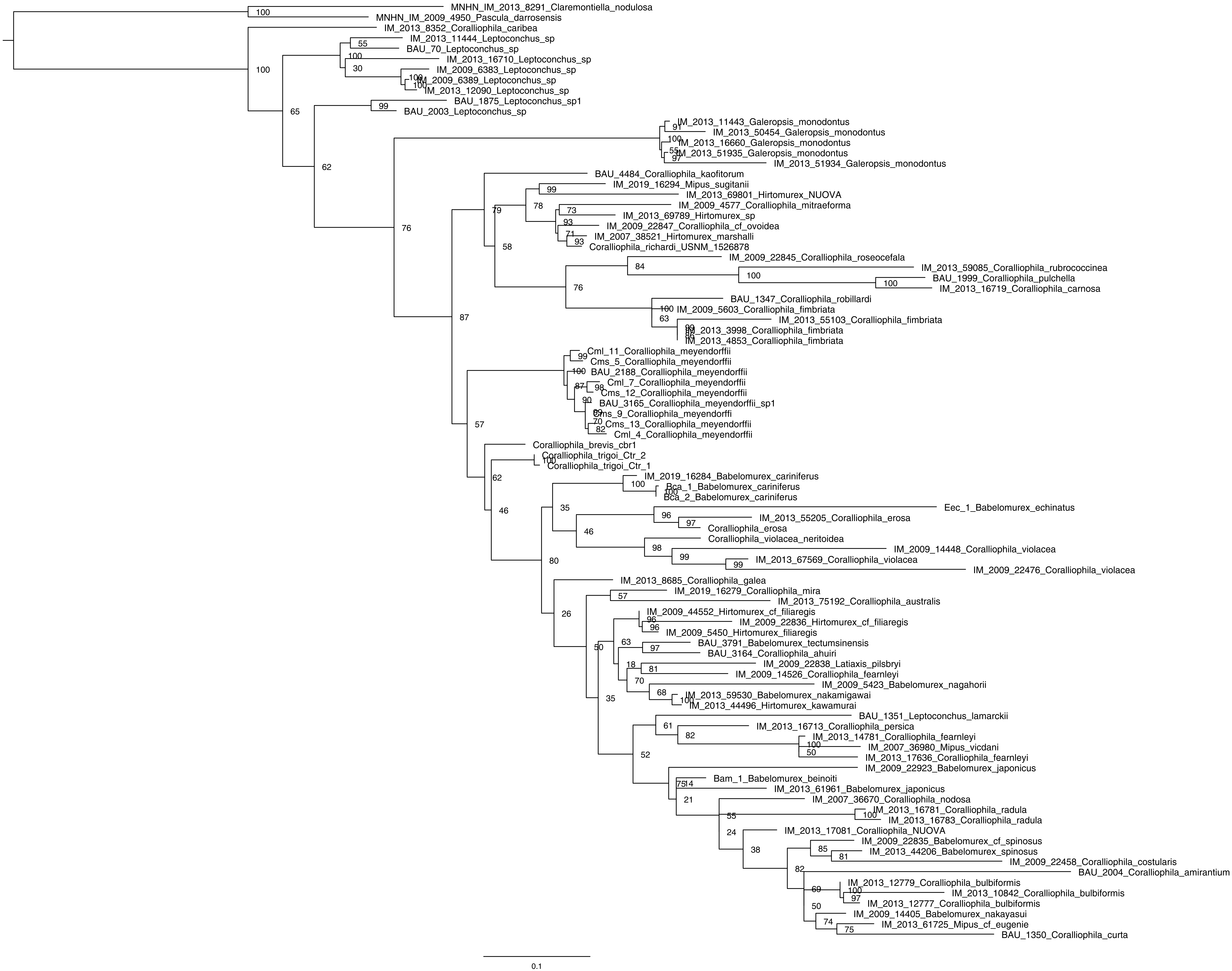

Figure S6. Bayesian phylogenetic inference on the COI-16S-ITS2 combined dataset. Numbers at nodes indicate posterior probability values.

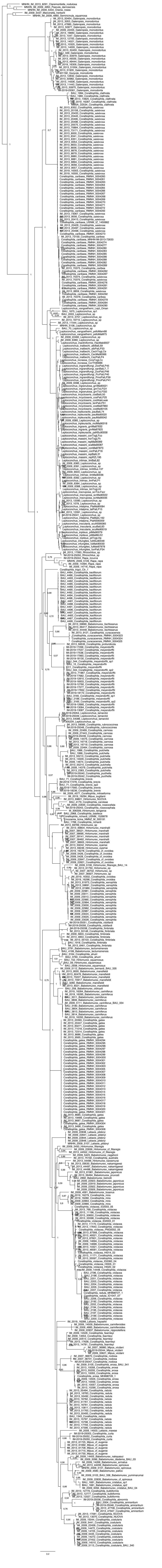

Figure S7. Bayesian phylogenetic inference on the COI dataset. Numbers at nodes indicate posterior probability values.

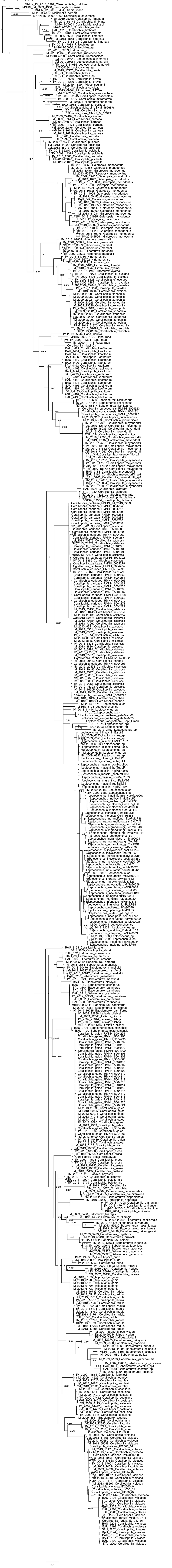

Figure S8. Bayesian phylogenetic inference on the 16S dataset. Numbers at nodes indicate posterior probability values.

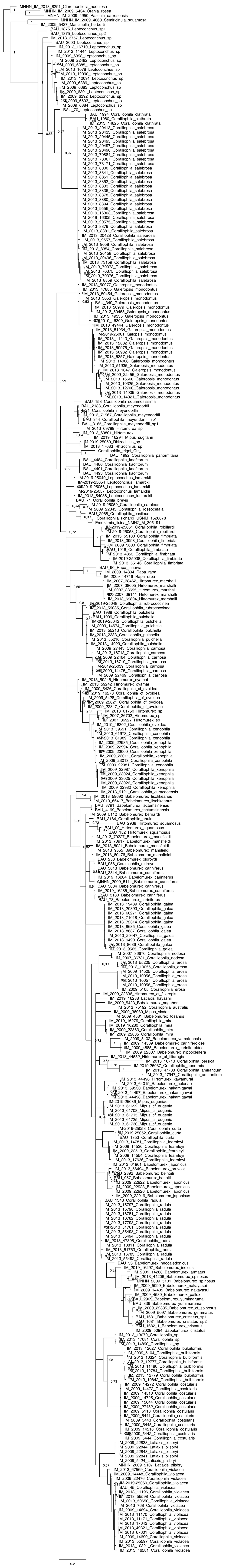

Figure S9. Bayesian phylogenetic inference on the ITS2 dataset. Numbers at nodes indicate posterior probability values.

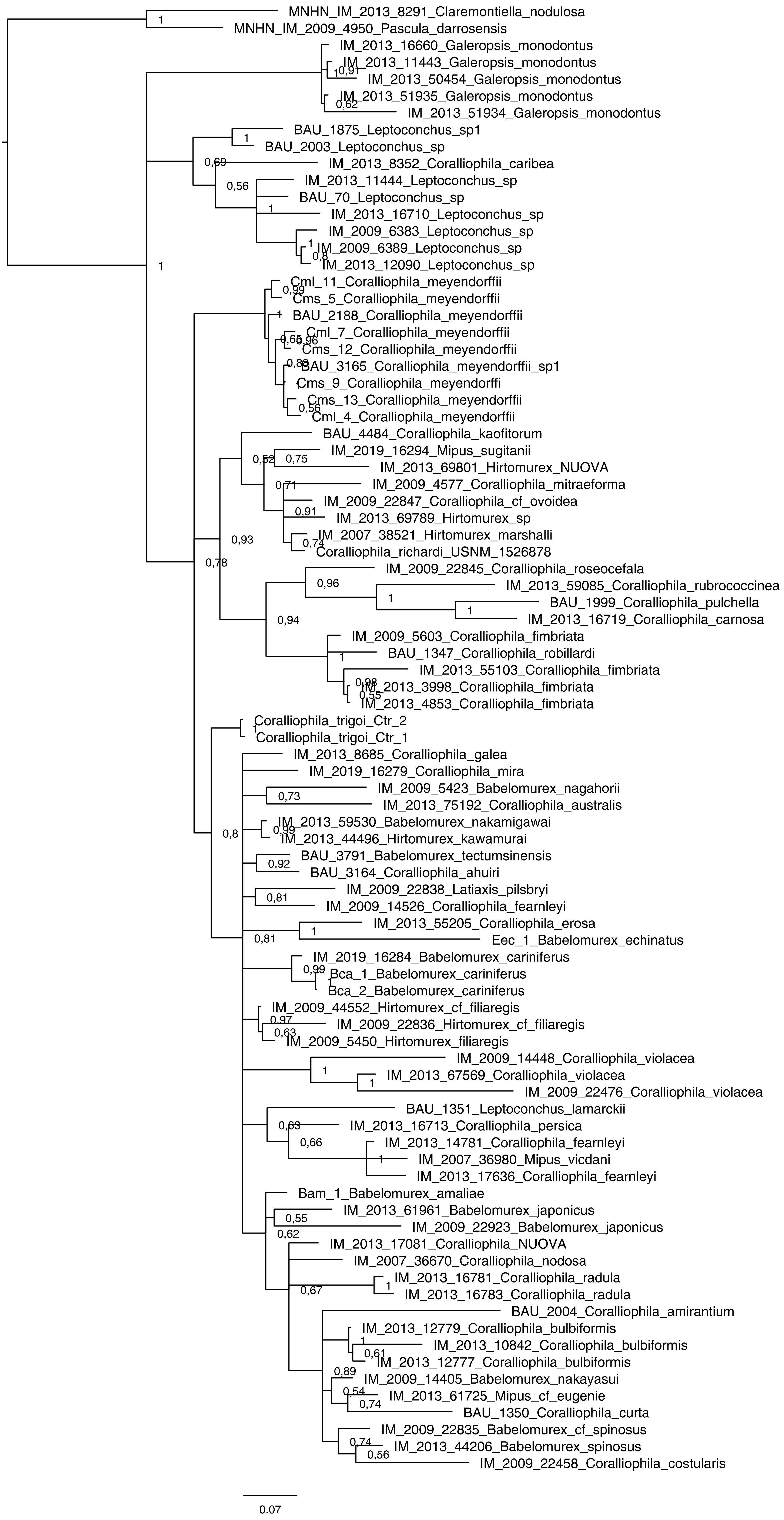

Supplement: Supplementary file 2 — (PDF 3293 KB) [file 338_2024_2537_MOESM2_ESM.pdf]
