# Supplementary material for: From coral reefs into the abyss: the evolution of corallivory in the Coralliophilinae (Neogastropoda, Muricidae)
Source: Coral Reefs. 2024 Aug 12;43(5):1285–302. doi: 10.1007/s00338-024-02537-1 (PMC11413129; doi:10.1007/s00338-024-02537-1)
Supplement: Supplementary file 3 — (PDF 78 KB) [file 338_2024_2537_MOESM3_ESM.pdf]

From coral reefs into the abyss: the evolution of corallivory in the Coralliophilinae (Neogastropoda, Muricidae)

*E. Nocella, G. Fassio, D. Zuccon, N. Puillandre, M.V. Modica, M. Oliverio

*Corresponding author: elisa.nocella@uniroma1.it
[1]Department of Biology and Biotechnologies "Charles Darwin", Sapienza University of Rome, Rome, Italy
[2]Department of Biology and Evolution of Marine Organisms, Stazione Zoologica Anton Dohrn, Napoli, Italy

Supplementary Material: tables captions

Table S1. List of gastropod samples used in this study, along with voucher registration numbers, collection localities, number of localities corresponding to orange points on the map (Fig. 2), depth range, cnidarian hosts and GenBank accession numbers for sequences; the names correspond to the morphological identification of each specimen (PSH); * indicates samples included in the combined dataset used for the macroevolutionary analysis; ° indicates identification of the coral host based on molecular data.

Table S2. List of cnidarians associated with coralliophiline samples used in this study, along with voucher registration numbers, corresponding gastropod, and GenBank accession numbers for sequences.

Table S3. Table 2. List of 63 species of Coralliophilinae associated with their respective cnidarian host family as documented in literature and this paper. N° of observations: number of coralliophiline samples actually reported as associated with the cnidarian host. † indicates data obtained from gastropod stomach barcoding; ° data obtained from coral tissue DNA amplification.

Supplementary Material: Part. 1

Molecular analysis and sequences alignment

For the gastropods, a 658 bp region of the mitochondrial cytochrome oxidase subunit 1, *cox1*, was amplified using either the universal primer pair LCO1490 and HCO2198 (Folmer et al. 1994) or the molluscan primer pair CoxAF and CoxAR (Colgan et al. 2003). When the amplification of this region failed, possibly due to DNA degradation, a shorter "minibarcode" region of ~350 bp was amplified, replacing the forward or the reverse primer with the internal primers mlCOIintF and mlCOIintR, respectively (Leray et al. 2013). A ~500 bp region of the mitochondrial *16S* rDNA gene was amplified with the primers 16SA (Palumbi 1996), and 16SB (Palumbi et al. 1991) or 16SH (Espiritu et al. 2001). A ~700 bp region of the nuclear *ITS2* rDNA gene was amplified with the primers 3d and 4r (Oliverio and Mariottini 2001). Sequences available in GenBank were also retrieved.
One mitochondrial and one nuclear marker were amplified for cnidarians. A ~700 bp region of the mitochondrial *16S* rDNA gene was amplified with the primers *16S*HA and *16S*HB (Cunningham and Buss, 1993) and a ~700 bp region of the nuclear *ITS2* rDNA gene was amplified with the primers 3d and 4r (Oliverio & Mariottini 2001); for the cnidarian DNA retrieved from the stomach contents, the ~700 bp region of the mitochondrial *16S* rDNA gene with 16SHA and 16SHB or a ~350 bp region of the nuclear second internal transcribed spacer (*ITS2*) with the primers 5.8S–436 and 28S-663 (Grajales et al. 2007) were amplified.
The PCR products were purified using ExoSAP-IT (79 µL H20, 20 µL rAPid Alkaline Phosphatase, 1 µL Exonuclease I) and both strands were sequenced at Macrogen Europe, or at Eurofins Scientific commercial service. In case more than one band was obtained, the single lower DNA band (corresponding to cnidarian DNA amplification) were excised from the gel and further purified with the Gel Extraction Kit (SIGMA). Excised bands were then sequenced.
The *cox1* sequences were aligned using the alignment algorithm of Geneious v. 11 and checked for stop codons, while *16S*, *28S* and *ITS2* sequences were aligned with MAFFT v.7 online (Katoh and Standley 2013; Katoh et al. 2019) using the E-INS-i algorithm.

Gastropod species delimitation

To evaluate the actual diversity of species within our collection of coralliophiline samples, we employed an integrative taxonomy methodology designed for extensive datasets, as outlined in Puillandre et al. (2012). In this process, primary species hypotheses (PSH) were formulated through morphological analysis of the shells, drawing on established taxonomic references (Oliverio et al. 2009a; Kosuge and Suzuki 1985). Shell features were examined for each specimen and integrated with published data.

The morphological species hypotheses were subsequently evaluated using molecular data. Initially, a genetic distance method was applied using the ASAP program (Assemble Species by Automatic Partitioning), which relies on an ascending hierarchical clustering algorithm to define species boundaries (Puillandre et al. 2021). ASAP analyses were conducted on the *cox1* ingroup dataset after removal of the 21 shorter minibarcode sequences, following Nocella et al. (2024). A first ASAP analysis was conducted to detect potential contaminations or individuals clearly misplaced based on morphological characteristics. Following the removal of contaminants and correction of any morphological discrepancies, a second ASAP analysis was carried out. The morphology-defined PSH were then compared to the partitions obtained with ASAP and discussed in the context of a phylogenetic tree derived from the combined genetic dataset (details provided below in the phylogenetic reconstruction section) and considering additional factors such as host associations, depth, and geographic range. This comprehensive assessment led to the proposal of Secondary Species Hypotheses (SSH).
